# Lymphocyte and dendritic cell response to a period of intensified training in young healthy humans and rodents: A systematic review and meta-analysis

**DOI:** 10.3389/fphys.2022.998925

**Published:** 2022-11-11

**Authors:** Carla Baker, John Hunt, Jessica Piasecki, John Hough

**Affiliations:** ^1^ SHAPE Research Centre, Department of Sport Science, Nottingham Trent University, Nottingham, United Kingdom; ^2^ Medical Technologies Innovation Facility, Nottingham Trent University, Nottingham, United Kingdom

**Keywords:** immune biomarkers, intensified exercise, dendritic cells, altered immunity, humans, rodents

## Abstract

**Background:** Intensified training coupled with sufficient recovery is required to improve athletic performance. A stress-recovery imbalance can lead to negative states of overtraining. Hormonal alterations associated with intensified training, such as blunted cortisol, may impair the immune response. Cortisol promotes the maturation and migration of dendritic cells which subsequently stimulate the T cell response. However, there are currently no clear reliable biomarkers to highlight the overtraining syndrome. This systematic review and meta-analysis examined the effect of intensified training on immune cells. Outcomes from this could provide insight into whether these markers may be used as an indicator of negative states of overtraining.

**Methods:** SPORTDiscus, PUBMED, Academic Search Complete, Scopus and Web of Science were searched until June 2022. Included articles reported on immune biomarkers relating to lymphocytes, dendritic cells, and cytokines before and after a period of intensified training, in humans and rodents, at rest and in response to exercise.

**Results:** 164 full texts were screened for eligibility. Across 57 eligible studies, 16 immune biomarkers were assessed. 7 were assessed at rest and in response to a bout of exercise, and 9 assessed at rest only. Included lymphocyte markers were CD3^+^, CD4^+^ and CD8^+^ T cell count, NK cell count, NK Cytolytic activity, lymphocyte proliferation and CD4/CD8 ratio. Dendritic cell markers examined were CD80, CD86, and MHC II expression. Cytokines included IL-1β, IL-2, IL-10, TNF-α and IFN-γ. A period of intensified training significantly decreased resting total lymphocyte (*d= −*0.57, 95% CI *−*0.30) and CD8^+^ T cell counts (*d= −*0.37, 95% CI *−*0.04), and unstimulated plasma IL-1β levels (*d= −*0.63, 95% CI *−*0.17). Resting dendritic cell CD86 expression significantly increased (*d =* 2.18, 95% CI 4.07). All other biomarkers remained unchanged.

**Conclusion:** Although some biomarkers alter after a period of intensified training, definitive immune biomarkers are limited. Specifically, due to low study numbers, further investigation into the dendritic cell response in human models is required.

## 1 Introduction

Overloading the body whilst preventing inadequate recovery is a necessary process implemented within an athletes’ training program to improve athletic performance (Whyte, 2006). If there is not an appropriate balance of stress and recovery, states of overtraining may occur. These states are functional overreaching (FOR), non-functional overreaching (NFOR) and the overtraining syndrome (OTS). When in a state of FOR a short-term decrement in performance may occur (Halson et al., 2002) but with sufficient recovery a “*supercompensatory*” effect on performance may be seen (Birrer et al., 2013). However, if recovery is not implemented at the appropriate moment, athletes may enter a state of NFOR (Kellmann et al., 2018) which could take weeks or months for full recovery to occur (Meeusen et al., 2013). If NFOR is left undiagnosed, and the training/recovery imbalance continues, athletes experience a heightened risk of suffering from the OTS, which can take months to years to fully recover (Meeusen et al., 2013). Symptoms of NFOR/OTS occur in individual (37%) and team (17%) sport athletes (Matos et al., 2011), with the incidence in an athletes’ career ranging from 30% to 60% (Morgan et al., 1987; Birrer et al., 2013). Despite the high incidence of states of overtraining, little progress has been made on establishing objective and reliable biomarkers for identifying when an athlete may be entering the various states of overtraining following periods of intensified training (Armstrong and VanHeest, 2002).

Cortisol is a hormone that is synthesised and released in response to physical and mental stress *via* the hypothalamic pituitary adrenal (HPA) axis. The HPA axis consists of the hypothalamus, pituitary gland and adrenal cortex (Guilliams and Edwards., 2010). During periods of intensified training, it has been reported that there is a blunting, by 72%, of the cortisol response to a short duration (30 min), high-intensity cycle test when comparing before to after an 11-day intensified training period (Hough et al., 2013). This disrupted functioning of the HPA axis following an intensified training period has previously been highlighted. Meeusen et al. (2004) examined the hormonal responses to an exercise stress test composed of two maximal cycle tests separated by 4 h resting recovery in well-trained athletes before and after a 10-day intensified training period. They reported a 118% and 73% reduction in the response of cortisol and adrenocorticotrophic hormone (ACTH; a precursor hormone to cortisol) in the athletes in response to the second maximal cycling bout after the 10-day training period compared to before the training period (Meeusen et al., 2004). Meeusen et al. (2010) also reported that athletes in a state of OTS (classified according to the duration and severity of symptoms and underperformance experienced) show little or no exercise-induced increases in ACTH in response to the second maximal exercise bout in their exercise stress test. This suggests that the exercise-induced response of the HPA hormones, specifically cortisol and ACTH, may be lowered following periods of intensified training.

Cortisol plays an important role in the anti-inflammatory response of the immune system to exercise by increasing the phagocytic potential of neutrophils and monocytes (Blannin et al., 1996; Ortega et al., 1996), supressing pro-inflammatory mediators such as reactive oxygen species (ROS) (Franchimont, 2004) and inducing lymphocytopenia (Okutsu et al., 2005). Lymphocytopenia refers to the lowering of lymphocytes in the blood, and most likely is a reflection of their increased migration into the tissues for increased immune-surveillance (Kruger et al., 2007). Therefore, a temporary dysfunctional HPA axis caused by a period of intensified training may lead, in part, to an impaired immune response during intensified exercise.

The impact of heavy periods of training on the immune system remains unclear, with some evidence suggesting a decline in immunity after repeated arduous exercise bouts (Walsh, 2019). As debated in Simpson et al. (2020), it is suggested that the reduced post-exercise immunosurveillance that occurs after prolonged (>5 days) and intensive (>60% $\dot{V}$ O_2max_) (Hoffman-Goetz et al., 1990) endurance training, in addition to the post-exercise decline in cytotoxic T cells (Steensberg et al., 2001) introduces a “window of opportunity” for infection. Repeated exposures to these acute declines in immunity bare additive negative consequences to infection risk (Pedersen and Ullum, 1994). In line with this, it has been reported that elite athletes that undergo heavy training regimes experience significantly higher episodes of upper respiratory tract infections (URIs) than recreational athletes (Spence et al., 2007), with a small proportion of athletes experiencing recurrent episodes at higher rates than the general population (Fricker et al., 2000). These recurring URIs have been associated with persistent fatigue that can hinder an athletes training (Reid et al., 2004). Moreover, it has been shown that elite endurance athletes prone to recurrent URIs i.e., more than 4 episodes per year, have an altered cytokine response, suggestive of impaired inflammatory regulation compared to healthy athletes (Cox et al., 2007). Similarly, a reversible defect in CD4^+^ T cell IFN-γ secretion, a cytokine known to affect illness severity and duration, has been associated with illness-prone athletes experiencing fatigue (Clancy et al., 2006). Furthermore, suppression of immune parameters can occur in elite athletes over years of training, which can result in reactivation of viruses (Gleeson et al., 2002; Reid et al., 2004). At a cellular level, studies have reported a reduced CD4+/CD8+ ratio in response to a 4 weeks strength training program involving progressive intensity increases each week from 75% to 85% heart rate maximum (HRmax) (Dongqing, 2013), a reduction in T cell proliferation immediately after a 30 min treadmill run at 80% $\dot{V}$ O_2max_ following a 3 weeks intensified training period (25% above normal training load) when compared to before the training (Verde T. J et al., 1992), and reduced natural killer (NK) cell cytotoxicity after 1 month of intense volleyball pre-season compared to before pre-season began (Suzui et al., 2004).

However, the “window of opportunity” theory is not accepted by all, with suggestions that reductions in immune cell function post-exercise could reflect the lowered number of immune cells in the circulation after exercise, which are redistributed into tissues for enhanced immunosurveillance at sites of infection risk (Kruger et al., 2007; Campbell et al., 2009). For example, Green et al. (2002) showed a significant decrease in lymphocyte proliferation–an important first step to create effector lymphocytes - after a 60 min, high-intensity run, but found no significant differences between the exercise and control groups when assessing lymphocyte proliferation in an NK cell depleted culture, or when adjusted per T cell. This suggests that the decreased proliferation found initially was likely due to an exercise-induced increase in NK cells within the sample, thus a reduction in the proportion of T cells that can be stimulated, rather than the exercise bout causing an actual reduction in T cell proliferation. Therefore, it is argued that studies reporting changes in immune cell function that coincide with changes in immune cell count cannot use lymphocytopenia as evidence for a decline in immunity. This is because the fall in cell number does not reflect mass apoptosis but a redistribution of highly functional T cells and NK cells from the bloodstream into the tissues and organs (Kruger et al., 2007; Campbell et al., 2009). This redistribution enhances the identification and eradication of tissue tumour cells; a clear benefit to the host. It has been shown that cancer cells incubated with exercised serum form less tumors when inoculated into mice (Hojman et al., 2018), and 4 weeks of voluntary wheel running prior to tumor cell inoculation reduced tumor growth by 61%, attributed to the redistribution of NK cells after exercise causing an increased infiltration of NK cells to tumor sites (Pedersen et al., 2016). Another commonly used measure of URI susceptibility in athletes is salivary immunoglobin A (sIgA). Although there are reports that the lowered sIgA seen with intense periods of training is associated with increased URIs in athletes (Fahlman and Engels, 2005), this has not been shown consistently (Antualpa et al., 2018; Gill et al., 2014; Pacque et al., 2007). Moreover, studies that do relate URI with decreased sIgA levels rarely consider confounding factors that may impact sIgA secretion and concentration, such as the profound intra-and inter-individual variation, likely due to oral health, psychological stress or sleep, and diurnal or seasonal-changes (Brandtzaeg, 2013). Finally, immune competency is also influenced by non-exercising factors, and without clinical confirmation that a URTI is present, symptoms could be due to allergy (Kennedy et al., 2016), or caused by variables such as psychological stress (Cohen et al., 1991), low energy availability (Bromley et al., 2018), or low sleep efficiency (Prather et al., 2015). Evidently the arguments for both an increased and reduced immune response post exercise are well supported and more definitive research is required to provide a firm conclusion.

The HPA axis is known to be involved in the regulation of important antigen presenting cells, involved in linking the innate and adaptive immune responses, known as dendritic cells (DC) (Liberman et al., 2018). Glucocorticoids, such as cortisol can regulate the maturation, survival, and migration toward the lymph nodes of DCs, but also can inhibit their immunogenic functions (Liberman et al., 2018). Cortisol itself has been shown to downregulate DC costimulatory molecules and dampen pro-inflammatory cytokine production, such as IL-6, IL-12 and TNF- α, which subsequently reduces the ability of the DCs to prime naïve CD8^+^ T cells (Elftman et al., 2007). Given the importance of these cytokines in orchestrating the immune response, the measure of cytokines, such as, TNF- α, IFN- γ and IL-1 β, as pro-inflammatory orchestrators of a type 1 immune response, and IL-10 and IL-2, as key anti-inflammatory immune-regulators, can act as a measure of immune function. Specifically, these cytokines are released from and also activate T cells and DCs (Blanco et al., 2007; Shaw et al., 2018).

Upon engulfing and processing an extracellular antigen, or degrading and processing intracellular antigens, DCs mature and gain T cell stimulatory capacity *via* antigen processing and upregulation of the major histocompatibility complex (MHC), costimulatory molecules (CD80/86) and cytokines (i.e., IL-12) (Wehr et al., 2019). The MHC is located on the surface of a DC and is loaded with peptide fragments from a pathogen, which it then presents to the T cell receptor for recognition (Guermonprez et al., 2002). CD80/86 are co-stimulatory molecules which bind to CD28 on the T cell to amplify the initial activating signals provided to the T cell receptors by the antigen loaded-MHC (Magee et al., 2012). Finally, the cytokines are required in order to drive the differentiation and proliferation of the T cells. All 3 of these signals are therefore required for T cell stimulation. The matured DCs then migrate towards the lymph nodes to present the antigen to T cells. An upregulation of MHC I complex is required for presentation of intracellular antigens to CD8^+^ T cells, whereas MHC II is loaded with extracellular antigens degraded *via* the endocytic pathway, for presentation to CD4^+^ T cells (British Society for Immunology, 2021). Cross presentation can also occur, meaning exogenous antigens can be presented by MHC I molecules. DCs also operate a bi-directional link with NK cells; a lymphocyte functioning within the innate immune system (Thomas and Yang, 2016). DCs can induce NK cell proliferation and cytotoxicity *via* the release of cytokines such as IL-12, IL-15 and IL-18 (Ferlazzo and Morandi, 2014). Conversely, NK cells can induce DC maturation *via* the secretion of IFN-γ and TNF-α (Moretta et al., 2006), and eliminate DCs that do not mature properly, a process known as ‘DC editing’, through engagement with the activating receptor NKp30 (Moretta et al., 2006). To our knowledge, there are currently four reports investigating the DC response to exercise training, indicating that after chronic exercise training in rats, DC function; as a measure of the expression of co-stimulatory molecules and MHC II receptors, and IL-12 production, required for T-cell stimulation, remains unchanged (CD80) (Liao et al., 2006; Chiang et al., 2007; Mackenzie et al., 2016; Fernandes et al., 2019), increased (CD86) (Chiang et al., 2007; Mackenzie et al., 2016) or unclear (MHC II) (Chiang et al., 2007; Mackenzie et al., 2016). Despite the known DC changes with HPA axis alterations, the lack of evidence surrounding DCs leaves the question of how DCs respond to periods of intensified training unanswered.

With evidence that DCs are in part regulated by the HPA axis, and the knowledge that the HPA axis response to exercise stress may be blunted following a period of intensified exercise, it is important to examine the impact that intensified exercise has on DCs. As these cells orchestrate the immune response, specifically, the direct nature of the relationship DCs have with both T lymphocytes and NK cells, it is logical to review evidence surrounding all three immune cells, providing further direction towards a conclusion in the response of the immune system to intensified training.

Therefore, the aim of this systematic review is to assess the current literature examining the effects of a period of intensified training on lymphocyte (T cells and NK cells) and DC number and function, in both humans and rodents. This review focuses on the normal impact of high intensity training due to the difficulties surrounding confirmation of NFOR/OTS diagnosis. However, heavy training is a factor involved in the establishment of NFR/OTS, and as such, any highlighted immune biomarkers could potentially indicate NFOR/OTS has occurred. The main purpose being to highlight areas already studied, indicate potential gaps requiring further investigation, and assess if there is scope for the future use of immune biomarkers in the diagnosis of overtraining.

## 2 Methods

This review conforms to the Preferred Reporting Items for Systematic Reviews and Meta-Analyses (PRISMA) guidelines (Moher et al., 2009) and was registered with PROSPERO international prospective register for systematic reviews (CRD42021248776; 21 May 2020).

### 2.1 Inclusion and exclusion criteria

To develop the inclusion and exclusion criteria for this review a consideration of Population, Intervention, Comparison and Outcome (PICO) was used (Richardson et al., 1995).

### 2.2 Eligibility criteria

#### 2.2.1 Population

Humans aged 18–50 years with a maximum oxygen uptake ( $\dot{V}$ O_2max_) of fair or higher (>38.5 ml kg^−1^. min^−1^) according to ACSM guidelines for cardiorespiratory fitness (ACSM, 2017) or Rodents aged 6 weeks—5 months were included in this review.

Human studies using females must have controlled for menstrual cycle to be included in the review. The menstrual cycle is known to impact certain elements of the immune system e.g. lowered CD4^+^ T cell numbers and increased type 1 cytokine production during the luteal phase compared to the follicular phase (Timmons et al., 2005; Oertelt-Prigione, 2012).

#### 2.2.2 Intervention

Studies must include an increased training load compared to their regular training load, completed over multiple days.

#### 2.2.3 Comparison

Studies included were required to have a comparative control. In human studies, participants were used as their own controls, comparing their pre-and post-training biomarker values. Where no pre-training values were given in rodent studies, the control group was used as a comparison.

#### 2.2.4 Outcome

Studies must have measured at least one immunological biomarker relating to lymphocytes, DC, or cytokines before and after a period of training. The immune biomarkers could be measured at rest, or in response to an acute bout of exercise; this will be referred to as “exercise-induced” and indicates that the biomarker was measured immediately after an acute exercise bout both before and after a period of intensified training. Data must have been presented as mean and standard deviation to allow the calculation of the standardised mean difference (SMD) of the change in biomarker from pre-to post-training. A minimum of two studies measuring the same biomarker, using the same measurement units, were required to include that biomarker in the meta-analysis component. Where possible, differing units of measurements were converted into the same “gold standard” units for comparison. If this was not possible, it was excluded from the meta-analysis.

### 2.3 Search strategy for identification of studies

A literature search was conducted in the following databases on 26 May 2021: SPORTDiscus, PUBMED, Academic Search Complete, Scopus and Web of Science. Databases were searched from inception up until May 2021 for articles published in English. In addition to database searches, reference lists of relevant studies were screened for eligible studies. The search was re-run in June 2022 to identify any additional articles meeting the inclusion criteria.

Titles, abstract and keywords were searched using the following search terms:

1) “chronic exercise*” OR “training volume” OR “intensified training” OR “exercise training” OR “overtrain*” OR “endurance training*” OR “physical education and training” OR “high intensity training” OR “chronic exercise training” OR “physical conditioning, animal*” OR “Physical exertion”
2) “lymphocyte function” OR “immune response” OR “dendritic cell function” OR “immune function” OR “dendritic cell” OR “myeloid” OR “plasmacytoid” OR “t cell*” OR “cd4*” OR “cd8*” OR “T helper” OR “T cytotoxic” OR “lymphocyte*” OR “NK cell” OR “natural killer cell” OR “cd56*” OR “T regulatory” OR “cd25*” OR “lymphocyte proliferation” OR “T cell proliferation” OR “CD80*” OR “CD86*” OR “cd80*/86*” OR “NK-cell” OR “NKCA” OR “Natural Killer cell cytotoxic activity” OR “killer cells, natural” OR “cytotoxicity, immunologic” OR “lymphocyte activation” OR “antigen presenting cell*” OR “dendritic cells” OR “genes, mhc class i” OR “genes, mhc class ii” OR “interleukin”
3) “athlete*” OR “Mice” OR “animals”
4) “elderly” OR “Cancer” OR “Elder” OR “older” OR “geriatric” OR “aged”
5) AND 2 AND 3 NOT 4.

### 2.4 Study selection

Articles retrieved through the systematic search were exported to ProQuest RefWorks, a reference management software (RefWorks 3.0, Pro-Quest LLC, Michigan U.S.), and further exported to Excel (Microsoft 365, Microsoft, Washington, United States ), whereby duplicates were removed and assessment for eligibility began. Two investigators (CB and JH) independently screened articles by title and abstract, and full text when necessary, against the inclusion criteria. Full texts from the eligible studies were then independently screened (CB and JH) for inclusion into the review.

### 2.5 Data extraction and management

Data extraction was conducted by one reviewer (CB) whereby the following data from all eligible articles were extracted into an Excel document: Title, publication details (year and author), participant characteristics (sex, age, number, $\dot{V}$ O_2max_, age), intensified training period details (mode and duration) and assessed biomarker information (biomarker assessed, and method and units of measurement). Pre- and post-training values were extracted for each relevant biomarker in the form of mean and standard deviation. Where appropriate data was not presented, the authors were emailed, and were allocated 4 weeks to reply. If no reply was received after 4 weeks, the study was excluded. Any variables included in the search string that did not have sufficient studies to perform a meta-analysis were not included in the results. Where figures were used displaying the mean and standard deviation, data was extracted by eye.

### 2.6 Risk of bias

Risk of bias was assessed by one reviewer (CB) and independently verified by one member of the review team (JH). Three Cochrane Collaboration tools were used for assessing risk of bias; ROBINS-1 for non-randomised controlled trials, ROB-2 for randomised controlled trials and ROB-2 (Crossover) for randomised crossover trials (Cochrane Collaboration 2021; Oxford, United Kingdom). Specific study components assessed for risk of bias using the ROBINS-1 tool included confounding, selection of participants, classification of intervention, deviations from intended interventions, missing data, measurement of outcomes and reporting of results. Study components assessed using the ROB-2 tool included the randomisation process, deviations from intended interventions, missing outcome data, measurement of outcomes and reporting of results. The ROB-2 crossover tool assessed the same components as the ROB-2 tool with the addition of carryover effects.

### 2.7 Statistical analysis

Inverse variance, random effects meta-analysis was then conducted on immune biomarker data in Review Manager Software (RevMan, Version 5.3, Cochrane Collaboration, Oxford, United Kingdom). Hedge’s *g* standardised mean difference (SMD) was calculated *via* the RevMan software (RevMan, Version 5.3, Cochrane Collaboration, Oxford, United Kingdom).

A separate meta-analysis was conducted for each biomarker where >2 studies measured the same biomarker using the same method and units of measurement. Human and rodent studies were analysed together for all biomarkers, apart from ‘lymphocyte proliferation’ due to human studies measuring peripheral blood lymphocytes, and rodent studies measuring spleenocytes. Effect sizes were classified based on the magnitude of change from pre to post intervention. Classifications included very small (0.01–0.19), small (0.20–0.49), moderate (0.50–0.79), large (0.80–1.19), very large (1.20–1.99) and huge (>2.0) (Cohen, 1988; Turner and Bernard, 2006; Sawilowsky, 2009). Statistical heterogeneity was determined using the I^2^ statistic; 0%–40% indicated non-important (low) heterogeneity, 40%–60% indicted moderate heterogeneity, 50%–75% indicated substantial heterogeneity and 75%–100% indicated considerable heterogeneity (Cochrane, 2021). All results were reported as Hedge’s *g* with 95% confidence intervals (CI). Additional sub-group analysis was conducted on resting immune cell count biomarkers based on the duration of intensified training periods i.e. ≤ 7 days, 8 days-2 weeks, 15 days- 4 weeks or >4 weeks.

## 3 Results

### 3.1 Risk of Bias

A complete analysis of ROB is displayed in Table 1. For studies assessed with the ROBINS-1 tool, bias in “selection of participants to the study” was deemed as “not applicable” (*n* = 3) or “Low” (*n* = 30) because most studies followed a group of athletes over time or assessed the same group of participants before and after a period of intensified training. The bias arising from participant awareness of intervention encapsulated in ROBINS-1 domain 6; bias in measurement of outcome, was judged as being negligible in most studies (*n* = 24). It is difficult to blind participants from intervention when intensified training is the independent variable and training loads were often monitored or implemented by the investigators themselves, so knowledge of intervention was necessary. It could be argued that as objective immune biomarkers were measured, results are unlikely to be affected by knowledge of intervention, especially in the rodent studies.

**TABLE 1:** Risk of Bias assessment of included studies using Cochranes ROB-2, ROBIN-2 Cross-Over and ROBINS-1 tools. ROB-2 and ROB-2 Cross-Over: Low (✓), Some concern (∼), High (X), Not enough Information (?). ROBINS-1: Low (✓), Moderate (∼), Serious (S), Critical (X), Not enough information (?).

**ROB-2**						
**Study**	**Domain 1**	**Domain 2**	**Domain 3**		**Domain 4**	**Domain 5**		**Overall ROB**
	**Risk of Bias arising from tde randomisation process**	**Risk of Bias due to deviations from tde intended interventions**	**Risk of Bias due to missing outcome data**	**Risk of Bias in measurement of tde outcome**		**Risk of Bias in selection of tde reported result**		
Croft et al. (2009)	✓	∼	✓		✓	✓		∼
Gholamnezhad et al. (2014)	✓	✓	✓		✓	✓		✓
Hack et al. (1997)	✓	✓	?		✓	✓		✓
Hasanli et al. (2021)	✓	✓	?		✓	✓		✓
Hoffman-Goetz. (1986)	✓	✓	?		✓	✓		∼
Hoffman-Goetz et al. (1988)	✓	✓	?		✓	✓		✓
Hwang et al. (2007)	✓	✓	?		✓	✓		✓
Kaufaman et a (1994)	✓	✓	✓		?	✓		✓
Koyama et al. (1998)	✓	✓	?		✓	✓		✓
Kwak (2006)	✓	✓	?		✓	✓		✓
Louis et al. (2016)	✓	✓	?		✓	✓		✓
Mackenzie et al. (2016)	✓	✓	?		✓	✓		✓
Mitchell et al. (1996)	✓	✓	✓		✓	✓		✓
Peijie et al. (2003)	✓	✓	✓		✓	✓		✓
Poffe et al. (2019)	✓	?	✓		✓	✓		✓
Sheyklouvand et al. (2018)	✓	✓	?		✓	✓		✓
Wang et al. (2011)	✓	∼	?		✓	✓		∼
Wang et al. (2011)	✓	∼	?		✓	✓		∼
Watson (1986)	∼	✓	X		✓	✓		x
Weng et al. (2013)	✓	✓	✓		✓	✓		✓
Zhang et al. (2019)	✓	?	✓		✓	✓		✓
**ROB-2 Cross-Over**							
**Study**	**Domain 1**	**Domain S**	**Domain 2**	**Domain 3**	**Domain 4**	**Domain 5**		**Overall ROB**
	**Risk of Bias arising from tde randomisation process**	**Risk of Bias arising from period and carryover effects**	**Risk of Bias due to deviations from tde intended interventions**	**Risk of Bias due to missing outcome data**	**Risk of Bias in measurement of tde outcome**	**Risk of Bias in selection of tde reported result**		
Li et al. (2013)	✓	✓	✓	✓	∼	✓		∼
Meyer et al. (2004)	∼	∼	✓	✓	✓	✓		∼
Pizza et al. (1995)	?	✓	∼	✓	✓	✓		∼
**ROBINS-1**								
**Study**	**Domain 1**	**Domain 2**	**Domain 3**	**Domain 4**	**Domain 5**	**Domain 6**	**Domain 7**	**Overall ROB**
	**Bias due to confounding**	**Bias in selection of participants into the study**	**Bias in classification of interventions**	**Bias due to deviations from intended interventions**	**Bias due to missing data**	**Bias in measurement of outcomes**	**Bias in selection of the reported result**	
Baj et al. (1994)	✓	✓	✓	✓	✓	✓	✓	✓
Baum et al. (1994)	∼	✓	✓	?	?	∼	✓	∼
Blank et al. (1994)	✓	✓	✓	✓	✓	✓	✓	✓
Borges et al. (2012)	∼	✓	✓	?	✓	∼	✓	∼
Borges et al. (2018)	✓	✓	✓	?	✓	✓	✓	✓
Bresciani et al. (2011)	✓	?	✓	?	?	∼	✓	?
Bury et al. (1998)	∼	?	✓	✓	✓	✓	✓	∼
Chiang et al. (2007)	✓	✓	✓	✓	✓	✓	✓	✓
Chung et al. (2021)	✓	✓	✓	✓	?	✓	✓	✓
Córdova Martinez et al. (2015)	✓	✓	✓	✓	∼	∼	✓	∼
Dongqing (2013)	✓	✓	✓	✓	?	✓	✓	∼
Dressendorfer et (2002)	✓	✓	✓	✓	✓	∼	✓	∼
Ferry et al. (1990)	✓	✓	?	?	?	✓	✓	?
Fry et al. (1992)	✓	?	?	?	?	∼	✓	?
Halson et al. (2003)	?	✓	✓	?	?	✓	✓	?
Heisterberg et al. (2013)	✓	✓	✓	?	✓	✓	✓	✓
Jurimae and Purge. (2021)	✓	✓	?	?	?	✓	✓	?
Kajiura et al. (1995)	✓	✓	✓	?	?	?	✓	?
Lancaster et al. (2004)	✓	✓	✓	?	✓	✓	?	✓
Leet al. al. (2021)	?	✓	✓	✓	?	✓	✓	✓
Main et al. (2010)	✓	✓	✓	?	?	∼	?	?
Mueller (2001)	✓	✓	✓	?	?	?	✓	?
Mujika et al. (1996)	✓	✓	✓	?	✓	✓	✓	✓
Ndon et al. (1992)	✓	✓	✓	?	∼	✓	✓	∼
Nickel et al. (2011)	?	✓	✓	✓	✓	✓	✓	✓
Peake et al. (2003)	✓	✓	✓	?	?	✓	✓	✓
Rebelo et al. (1998)	✓	✓	✓	?	?	✓	✓	✓
Ronsen et al. (2001)	✓	✓	✓	✓	∼	✓	∼	∼
Shing et al. (2007)	✓	✓	✓	?	?	✓	✓	✓
Smith and Myburgh. (2006)	✓	✓	✓	?	?	✓	✓	✓
Tanimura et al. (2009)	✓	✓	✓	?	?	✓	✓	✓
Verde T et al. (1992)	✓	✓	✓	?	?	✓	✓	✓
Witard et al. (2012)	**✓**	**✓**	**✓**	**?**	**?**	**✓**	**✓**	**✓**

Despite this, it has been suggested that anticipatory stress may cause alterations to the immune system, such as decreased lymphocyte counts and reduced lymphocyte proliferation (Ironson et al., 1990; Ader and Cohen, 1991; Lekander, 2001). However, studies investigating this phenomenon tend to use the anticipatory stress surrounding major life events such as cancer patients waiting for chemotherapy treatment (Lekander, 2001), and homosexual men waiting for HIV test results (Ironson et al., 1990). The evoked stress response caused by such serious events could be deemed as incomparable to the anticipation of undertaking exercise, especially when undertaken by trained athletes. Therefore, whilst we acknowledge that the anticipation of undertaking exercise may elicit a stress response to some extent, perhaps more so in untrained personnel, it is an unavoidable, and potentially non-significant bias. It is impossible to know the true effect anticipatory stress may have on the measured immunological outcomes without studies undertaking measures of stress scores.

Bias due to missing outcome data and attrition rate was mainly low (*n* = 22) or unclear (*n* = 31) in most studies, mainly because no information regarding excluded participants or reasons for missing data were highlighted. Only one study (Watson, 1986) was rated as “high” for bias due to missing data as table 4 only included *n* = 5 results for the placebo group’s % T lymphocytes, when the placebo group consisted of 15 participants. A ‘moderate’ rating for bias due to missing outcome data was given for Córdova Martinez et al. (2015), as although they stated blood samples were collected before and after each stage of the cycling competition, unlike the other blood markers, only pre and post competition values were reported for cytokines. Ronsen et al. (2001) and Ndon et al. (1992) were also rated as “moderate” for bias due to missing outcome data as participants with incomplete data sets were still included in the final analysis (Ronsen et al., 2001) and participants were excluded from analysis by the investigators after final outcome measures were taken as they were perceived to be overtrained (Ndon et al., 1992).

### 3.2 Study outcomes

**FIGURE 1:**
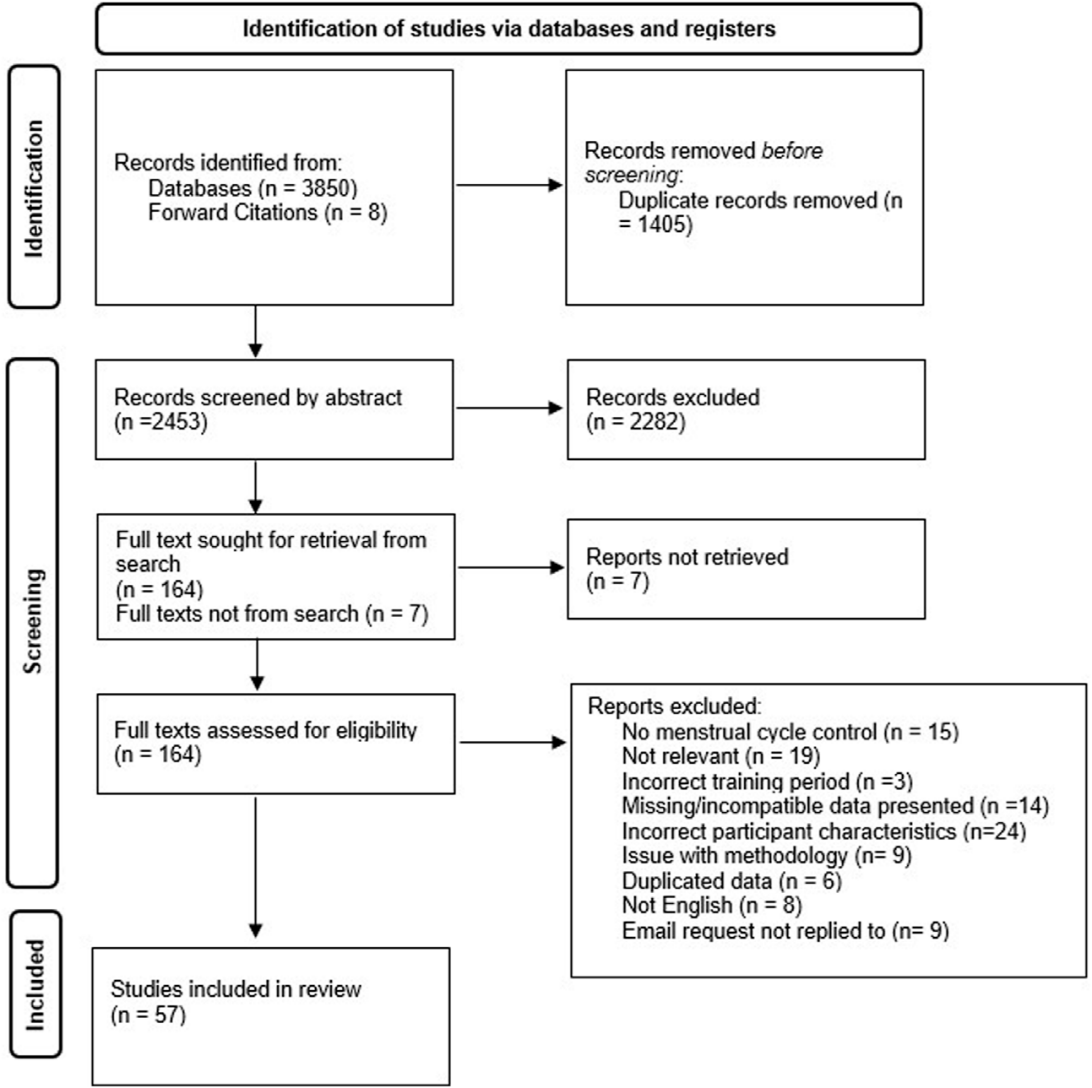
Systematic literature search PRISMA (Preferred Reporting Items for Systematic Reviews and Meta-Analyses) flow chart.

(Figure 1) Across the 57 included studies (Table 2), the variables used to assess immune cell changes included immune cell counts (Ferry et al., 1990; Fry et al., 1992; Ndon et al., 1992; Baj et al., 1994; Baum et al., 1994; Pizza et al., 1995; Mitchell et al., 1996; Mujika et al., 1996; Hack et al., 1997; Bury et al., 1998; Rebelo et al., 1998; Mueller, 2001; Ronsen et al., 2001; Dressendorfer et al., 2002; Halson et al., 2003; Peake et al., 2003; Lancaster et al., 2004; Meyer et al., 2004; Smith and Myburgh, 2006; Shing et al., 2007; Tanimura et al., 2009; Bresciani et al., 2011; Wang et al., 2011; Wang et al., 2011; Borges et al., 2012; Witard et al., 2012; Heisterberg et al., 2013; Li et al., 2013; Weng et al., 2013; Louis et al., 2016; Sheyklouvand et al., 2018; Poffe et al., 2019; Chung et al., 2021; Leal et al., 2021), lymphocyte proliferation (Hoffman-Goetz, 1986; Watson, 1986; Hoffman-Goetz et al., 1988; Verde T et al., 1992; Mitchell et al., 1996; Bury et al., 1998; Koyama et al., 1998; Peake et al., 2003; Peijie et al., 2003; Kwak, 2006; Hwang et al., 2007), CD4/CD8 ratio (Ferry et al., 1990; Fry et al., 1992; Verde T et al., 1992; Blank et al., 1994; Kaufman et al., 1994; Kajiura et al., 1995; Pizza et al., 1995; Hack et al., 1997; Mueller, 2001; Dressendorfer et al., 2002; Smith and Myburgh, 2006; Shing et al., 2007; Wang et al., 2011; Dongqing, 2013; Li et al., 2013; Weng et al., 2013; Poffe et al., 2019; Zhang et al., 2019; Leal et al., 2021), cytokine secretion (Dressendorfer et al., 2002; Halson et al., 2003; Shing et al., 2007; Croft et al., 2009; Main et al., 2010; Bresciani et al., 2011; Nickel et al., 2011; Gholamnezhad et al., 2014; Córdova Martinez et al., 2015; Borges et al., 2018; Hasanli et al., 2021; Juirmae and Purge, 2021), dendritic cell co-stimulatory molecule and MHC II expression (Chiang et al., 2007; Mackenzie et al., 2016) and NK Cytolytic activity (Watson, 1986; Bury et al., 1998; Shing et al., 2007).

**TABLE 2:** Return of relevant studies from the systematic search. Studies in Bold Italics were eligible for inclusion from the systematic search, but were not suitable for inclusion into the meta-analysis.

Study	Participants	Age	$\dot{V}$ O_2max_	Training status	Intensified training	Duration	Measurement method	Biomarker
***Anomasiri et al. (2002)***	***Humans (Males)***	***21.1 ± 0.44***	***40.2 ± 8.7***	***Trained***	***Military Training, 5d.wk***	***8 weeks***	***Flow Cytometry***	***CD3+, CD4+, CD8+, NK cell, lymphocyte counts***
Baj et al. (1994)	Humans (males)	21 ± 1.5	74.0 ± 1.4	Trained	Cycling training and competition; 500 km.wk training & 12,000 km during competition	24 weeks	Flow cytometry	Lymphocyte, CD3+, CD4+, CD8+ and NK cell counts, CD3/CD4 daysRatio, lymphocyte proliferatio^+^, IL-^+^
Baum et al. (1994)	Humans (males)	20.8 ± 3	No Information	Trained	Training 3 phases: endurance runs (60–160 km.wk), 8 weeks anaerobic km wke (intensive training- submax and max runs, uphill runs) & competition phase. (Pre and post phase 2 used)	8 weeks	Flow cytometry	Lymphocyte, CD3+, CD4+, CD8+ Counts
Blank et al. (1994)	Rodents (females)	8–10 weeks	Not applicable	Not applicable	Treadmill running 60 min.d, 5 d.wk at 12 m/min, 8 degree gradient	10 weeks	Automated cell counter	CD4/CD8 Ratio, NK cell, CD4+ andCD8+ counts
***Blank et al. (1997)***	***Rodents (females***	***9–10 + weeks***	***Not applicable***	***Not applicable***	***Treadmill running 60 min.d, 5 d.wk at 12 m/min, 8 degree gradient***	***10 weeks***	***51Cr-release assay in lytic units and Flow cytometry.***	***Spleen NK cell, CD4+, CD8+ counts and NK cytolytic activity.***
Borges et al. (2012)	Humans (males)	22 ± 4.2	61.2 ± 5.5	Trained	Kayak season (t0-t2 timepoint used)	26 Weeks	Automatic cell counter.	Lymphocyte count.
Borges° (2018)	Humans (males)	22 ± 4.3	61.2 ± 5.5	Trained	11 weeks high volume, 5 weeks high intensity; (kayaking, running, swimming & strenth).	26 Weeks	ELISA (unstimulated)	TNF- ± α , IFN- γ , IL-1 β .
Bresciani et al. (2011)	Humans (males)	22.3 ± 1.4	45.2 ± 2.3	Recreationaly Active	Running 3d.wk starting at 40, 30 ,30 min per session and increasing weekly volume by 5 mins each session every week. (T1-13 used). Intensity started at 42.5% and increased to 80% TRIMP.	9 weeks	ELSA (unstmulated)nd Automatic cell counter	Resting Lymphocyte ± count, T ± F- α
Bury et al. (1998)	Humans (males)	24.2 ± 2.6	62.8 ± 4.0	Trained	Football season	40 weeks	Resting PHA stimulated labelled thymidine incorporation via liquid scintillation (proliferation). Immunofluorescent staining and microscope (count)	Lymphocyte, CD4+, CD8+, CD56+ Counts, lymphocyte proliferation.
***Cardoso et al (2018)***	***Rod(males)***	***6–8 weeks***	***Not applicable***	***Not applicable***	***30 mins swimming d***	***15 days***	***ELISA (unstimulated)***	***IL-10, IL-1 β , TNF- α***
Chiang et al. (2007)	Rodents (males)	9 weeks	Not applicable	Not applicable	Treadmill endurance tranining 6d.wk^+^(progressively increased from 10 mmin to 25 m/min and 5 min to 30 min sessions)	5 weeks	LPS stimulated, measured via. minw cytometry	BMD MHC II, Myeloid DC CD80 and CD86, DC IL12
Chung et al. (2021)	Rodents (males)	6 weeks	Not applicable	Not applicable	treadmill running; 15 m/min at a 5% day scope increasing by 3 m.min every 10 min until exhanstion. 1 session first 4 weeks and 2 sessions for last 4 week 4 hr rest between sessions in last 4 weeks.	8 weeks	Autmoated cell counter, ELISA (Unstimualted exercise induced)	Lymphocyte count, IL- β
Córdova Martinez et al. (2015)	Humans (males)	20.03 ± 0.9	73.2 ± 6.7	untrained	460 km cycling: 4 stages (Basal and post 3rd stage used)	3 days	ELISA (unstimulaed)	TNF- α , IFN- γ , 1L-1B
Croft et al. (209)	Humans (males)	20 ± 1	55.9 ± 6.8	untrained	High intensity interval running 4xwk: 10 mins at 70% $\dot{V}$ O_2max_ , 3 × 5 min at 90%, 1.5 min at 50%, then 10 ± min cooldown at 70%	6 weeks	Unstimulated Bead assay (flow cytometry)	TNF- α
Dongqing (2013)	Humans (males)	20.2 ± 1.3	No Inforation	Trained	Weightlifting; progressive incr ± ase each week@ 75%, 80%, 80%, 85% HRmax, 5.5 d.wk.	4 weeks	Fluo minnt double labeling × method using Imm noassay	Resting CD4, CD8+ counts, CD4/CD8 Ratio
***Dos Santos Cunha et al. (2004)***	***Rodents ((males)***	***6–8 weeks***	***Not applicable***	***Not applicable***	***Treadmill running, 5 d.wk at 60%–65%*** $\dot{V}$ ***O2max***	***5 weeks***	***CON A stimulated proliferation, Thymidine incor o wkon measured by liquid scintillation (dpm) and ELISA (PHA stimulated).***	***Resting and exercise induced spleen Lymphocyte proliferation, IL-2, IL-10 and TNF- α***
Dressendorfer et al. (2002)	Humans (males)	24.4 ± 2.1	59.3 ± 5.0	trained	HIIT at 100%HRmax 4d.wk on a bike, plus one wind tunnel cycle and one weight session per week. (Baseline to end of I phase used)	6.5 weeks	Flow cytometry.	Lymphocyte count Resting: CD3+, CD8+, CD4+ Counts. Exercising: CD3+, CD8+, CD4+ Counts
***Fernandes et al. (2019)***	***Rodents ((males)***	***6–8 weeks***	***Not applicable***	***Not applicable***	***Treadmill running 1 hr.d at 50% average max speed.***	***5 weeks***	***Flow cytometry.***	***pDC and mDC count, CD80 and)CD86 expression of lung ad lymph***
Ferry et al. (1990)	Humans (males)	20.1±2.9	63.2 ± 4.3	trained	Across a cycling training cycle	20 weeks	Flow cytometry	Resting: Lymphocyte, CD8+, CD4+, CD56+ Counts Exercising: Lymphocyte, CD8+, CD4+, CD56+ Counts. CD4/CD8 Ratio
***Fery et al. (1992)***	***Rodents (males)***	***12 weeks***	***Not applicable***	***Not applicable***	***Treadmill running 6(d.wk,)duration increased from 30–60 min and speed from 20–30 m.min***	***4 weeks***	***Flow cytometry***	***Spleen CD4+ (resting and +xerci+e) and+CD8+ (resting) counts***
Fry et al. (1992)	Humans (males)	3.6 ± 3.5 (No body mass (kg) provided to convert to ml.kg^-1^.min^-1^)	3.71 ± 0.14 (L.min^-1^)	trained	Army training: 10 d treadmill intervals 2x day (15 x1 min exercise period 2 mins rest in AM, PM= 10 × 1 min intervals 1 min rest), 5 dactive recovery (Day1–10 used).	10 days	Flow cytometry	Lymphocyte, CD3+, CD8+, CD4+ CD56+ Counts, CD4/CD8 ratio, lymphocyte proliferation.
Gholamnezhad et al. (2014)	Rodents (males)	6–8 weeks	Not applicable	Not applicable	Treadmill running at 25 m.min, 60 min.d, 6 d.wk	11 weeks	ELISA (unstimulated)	IL-10, IFN- γ , TNF- α
Hacket al. (1997)	Humans (males)	23.4 ± 0.8	No Information	untrained	Anaerobicr, 60 min sessions 3 d.wk 2x sprint sessions (90%–110% $\dot{V}$ O_2max_ with 5–8 min recovery; 5 × 80-300 m), 1 × 60 min weights session	8 weeks	Automated cell counter and flow cytometry	Lymphocyte, CD3+, CD4+, C8+ and CD4+CD45RA+ counts, CD4/CD8 Ratio
Halson et al. (2003)	Humans (males)	21.1 ±3.0	58.0 ± 1.7	trained	2 week normal cycling training, 2 week– intensified cycling eriod (7 d.wk 150% normal load)	4 weeks	ELISA (unstimulated) and flow cytometry	Lymphocyte count, TNF- α
Hasanli et al.^.^(2021)	Rodents (male)	10 weeks	Not applicable	Not applicable	Treadmill running; 5 d.wk at 15 m.min in week 1, increasing tso 25 m.min by week 8. Duration started at 60 min.session in week days and increased to 60 min in week 5.	8 weeks	ELISA (unstimulated)	IFN- γ
Heisterberg et al., (2013)	Humans (males)	26.3 ± 1.1	62.5	trained	Professional soccer season (5–8 x wk, 1.5- 2 hr. session days)	24 weeks	Automated cell counter	Lymphocyte count
Weng et al. (2013)	Rodents (males)	12 weeks	Not applicable	Not applicable	Treadmill runing at 28 m.min, 6 d. wk at gradient 8 degree	6 weeks	CON A stimulated thymidine incorporation via liquid scintillation (CPM)	Spleen–lymphocyte –oliferation
Hoffman-Goetz et al. (1988)	Rodents (males)	8 weeks	Not applicable	Not applicable	Treadmill running (2 wks; 12-30 m-min, 0-8 degree gradient, 30 min. d, 5 d.wk and 6 wks; 30 m.min, 8 degree gradient, 20min.d, 5 d.wk)	8 weeks	LPS and PW*via*timulated thymidine incorporation via liquid scintillation (cpm)	Lymphocyte proliferation
***Hoffman-Goetz et al. (1990)***	***Humans (males)***	***24.5 ± 0.9***	***46.4 ± 6.4***	***untrained***	***Cyclin–30 m*** $\dot{V}$ ***O***_***2ma–***_ ***, 1 hr.d***	***5 days***	***Flow cytometry***	***Resting and exercising CD3+, CD4+, CD8+ and NK cell counts***
Hwang et al. (2007)	Rodents (males)	6 weeks	Not applicable	Not applicable	Swimming 30 min.d, 5 d.wk in week 1, then extended by 10 min.d, up to 60 min.d, 5 d.wk	10 weeks	CON A and LPS induced thymidine incorporation via liquid scintillation (cpm)	Spleen lymphocyte proliferation
Jurimae and Purge (2021)	Humans (males)	25.0 ± 6.5	64.0 ± 3.5	trained	Rowing training, starting at 11.6 ± 1.4 hr.wk and increasing to 18.4 ± 1 hr.wk	24 weeks	ELISA (unstimulated)	IFN- γ , TNF- α , IL-1 β , IL-2
Kajiura et al. (1995)	Humans (males)	20.2 ± 1.8	60.1 ± 5.2	trained	High volume, high intensity running phase (100% increase in normal running load, with 1000 m intervals at 95%–100% $\dot{V}$ O_2max_ ever other day)	10 days	Flow cytomet ± y	Resting and exercising CD4+ count, CD4/CD8ratio
Kafman et l. (1994)	Rodents (males)	6 weeks	Not applicable	Not applicable	Swimming 15 min intervals, increased over 2.5 wks to 2 hr.d 5 d.wk, with an additional 1.5 wk at 2 hr.d 5 m.wk	4 weeks	Flow–cytometry	Spleen CD4/CD8 ratio
***Kilgore et al. (2002)***	***Humans (males)***	***28.3 ± 6.3***	***No Information***	***trained***	***Weightlifting; 57%–90% 1RM rang(ng fr)m 3–5 d.wk***	***6 weeks***	***Automated cell counter***	***Lymphocyte count***
Koyama et al. (1998)	Rodents (males)	7 days	Not applicable	Not applicable	Progressive wheel running; 1 wk (60–120 min.d), 3 wks (120 min.d, 6d.wk), average distance increased from 1500–2500 m.d	4 weeks	CON A induced thymidine incorporation via liquid scintillation (cpm)	Peripheral lymphocyte proliferation
Kwak (2006)	Rodents (males)	6 weeks	Not applicable	Not applicable	Swimming 1 week; 30 min.d, increased by 10 min.wk up to 60 min.d	1 days weeks	CON A and LPS induce thymidine incorporation via liquid scintillation (cpm)	Spleen lymphoc*via* proliferation
Lancaster et al. (2004)	Humans (males)	30 ± 2	60.6 ± 1.5	trained	Cycling training every day at 150% normal volume with $\dot{V}$ O_2max_ tests before and after.	6 days	Flow cytometry and geometric mean fluorescence intensity	CD3+, CD4+, CD8+, *via*+CD45RO+, CD8+CD45RO+ and lymphocyte counts, IFN- γ
Leal et al. (2021)	Humans (males)	21 ± 5	59 ± 6	untrained	Running session repeated in the order of; 90 min continuous treadmill; 70 min @55% v $\dot{V}$ O_2max_ & 20 mn @75% v $\dot{V}$ O_2max_; 5 km TT; 70 min treadmill at 12 RPE (borg) for 30 mins, 13 RPE for 3 mins &15 RP for 10 mins.	12 days	Flow cytometry and Automated cell counter	CD4+, CD3+, CD8+, NK cell counts, CD4/CD8 ratio, DC CD11c Expression
***Leandro et al. (2006)***	***Rodents (male)***	***No Information***	***Not applicable***	***Not applicabe***	***Treadmill running 5 d.wk, 60 min.d a(70%*** $\dot{V}$ ***O***_***2max***_	***8 weeks***	***CON A induced minymidine incorporation via liquid scintillation***	***Lymphocyte proliferation***
Li et al.^.^(2013)	Humans (male)	19.2 ± 1.6	No Information	trained	Military training	1 week	Flow cytometry	CD4/CD8 ratio, Resting NK cell, CD3+, CD4+ count
***Liao et al. (2006)***	***Rodents (male)***	***6–8 weeks***	***Not applicable***	***Not applicable***	***Treadmill running 6 d.wk, timing increased from 15-35 min.d, speed increased from 10-25 m.m(n. 2) incline increase in last week. 30% intensity every 3rd day of each week.***	***5 weeks***	***Flow cytometry***	***mDC CD80 and +D86 e+pression, mDC count***
Louis et al. (2016)	Humans (male)	31.0 ± 4.7	58.7 ± 5.6	trained	6 sessions over 4 consecutive days, 10-15 h.wk; High intensity afternoon session (8 × 5 min cycling @85% MAP and 6 × 5 min running at 10 km intensity); low intensity the next morning (60 min cycle at 65% MAP). Light sessions 3 d.wk.	3 weeks	Automated cell counter	Lymphocyte count
Mackenzie et al. (2016)	Rodents (males)	6–8 weeks	Not applicable	Not applicable	Treadmill running at 60% m × x velocity, 1hr.d, 5d.wk	4 weeks	Flow cytometry	Bone marrow dendritic cell count
Main et al. (2010)	Humans (males)	26.6 ± 4.1	65.0 ± 34 days	trined	14 sessions. Wk; 7d.wk^-1^ (10 x rowing, the rest weights, running and ergometer). 24 h.–k^-1^,80% endurance based, 20% at LT and max sprint efforts	8 weeks	low cytometry (unstimulated)	IL-1β , TNF- α , IL-12p70, IL-10
Meyer et al. (2004)	Humans (males)	24.8 ± 3.8	68.4 ± 10.0	trained	Cycling training 20 h.wk^-1^ (40% i days crease from normal training volume)	13 days	Flow cytometry	Lymphocyte and NK cell counts
Mitchell et al., 1996	Humans (males)	23.4 ± 7.0	40.4 ± 0.1	untrained	Cycle ergoeter; 30 min.d^-1^, 2 d.wk^-1^ at 75% $\dot{V}$ O_2max_	12 weeks	PHA and PWM induced thymidine incorporation via liquid scintil ation	Lymphocyte count, lymphocyte proliferation
Mueller, 2001	Humans (males)	25.8—37.9	73.7 ± 4.7 (Ex) 44.8 ± 5.2 (Con)	trained	Season of cross country endurance skiing	8 weeks	ELISA, flow cytometry	Lymphocyte, CD3+, CD4+, CD8+ counts, CD4/CD8 ratio, IFN- γ , IL-10, IL-12
Mujika et al. (1996)	Humans (males)	21.1 ± 3.4	No Information	trained	Swimming season; 12 weeks traini– 4 weeks taper	16 weeks	Automated cell counter	Lymphocyte count
Ndon et al. (1992)	Humans (males)	25.6 ± 2.6	67.9 ± 2.3	trained	Cycling, swimming, running and weights training; 150% normal training duration	4 weeks	Automated cell counter	Lymphocyte count
Nickel et al. (2011)	Humans (males)	40 ± 7	No Information	trained	Continuous aerobic running and interval training, gradual increase in training and intensity (week 1: 38 ± 1 km.wk^-1^ – week 10: 54 ± 2 km.wk )	10 weeks	ELISA (unstimulated)	TNF- α
Peake et al. (2003)	Humans (males)	28 ± 7	No Information	trained	16% increase normal running training volume; 104±48 km average distance	4 weeks	Flow cytometer, CON A and PWM induced thymidine incorporation via liquid scintill ± t ion	Lymphocyte, CD3+, CD4+, CD8+, NK cell counts, lymphocyte proliferation
Peijie et al. (2003)	Rodents (males)	6 weeks	Not applicable	Not applicable	Swimming; Week 1 (1.4 m.s^-1^ for ± 0 in) increased by 5 mins.d^-1^ until 120 min.d^-1^. intensity increased to 1.6 m.s^-1^ at week 2 *via* 1.8 m.s^-1^ at week 5.	6 weeks	CON A induced thymidine incorporation via liquid scintillation	Spleen lymphocyte proliferation
Pizza et al. (1995)	Humans (males)	34.8 ± 7.6	65.1 ± 4.9	trained	200% increased running training min than normal	10 days	Flow cytometry	Lymphocyte, CD +, CD3+, CD8+ and NK ce l counts, CD4/C8 Ratio
Poffe et al. (2019)	Humans (males)	21.2 ± 2.9	55.3 ± 6.1	trained	Cycling 6d.wk^-1^; HIIT (30 s max sprint 100 rpm, 4.5 min active recovery at 50 W; sprints increased from 4–6 over 3 weeks); intermittent endurance (5 × 6 min 100-110% av. PO, 8 min 55%–85%^+^recov^+^ry periods); constant load endurance (70%–95% av. PO 30 min TT for 60-150 min)	3 weeks	Flow cytometry	CD3+, CD4+, CD days+ counts, CD4/CD8 ratio
Rebelo et al. (1998)	Humans (males)	26.3 ± 3.7	No Information	trained	Across an entire Portuguese football season	44 weeks	Flow cytometry	Lymphoc–tes, CD3+, CD4+ counts
Ronsen et al. (2001)	Humans–(males)	21—29	7—82	trained	Nordic skiing season	8 weeks	Automate^+^ cell^+^counter	Lymphocyte count
Sheyklouvand e (2018)	Humans (males)	24 ± 3	No Information	trained	Canoe paddling based HIIT 3d.wk, variable intensity (6 × 60 s at 100%, 110%, 120%, 130^+^, 130^+^, 130%, 120%, 130%, 100% v $\dot{V}$ O_2peak_ ) across 9 sessions	3 weeks	Automated cell counter	Lymphocyte count
Shing et al. (2007)	Humans (males)	27 ± 2	69.3 ± 1.3	trained	High intensity cycle training >VT. Day 1: 20 × 1 min at PPO, 2 min days covery at 50 W; Day 2: 60 min × at 100%VT; Day 3: 12 × 30 s sprints at 175% PPO, 4.5min at 50 W recovery; ay 4: 30 mins at 80%VT, 45 min at 100%VT; Day 5: 40 min TT	5 days	Flow cytometry	Lymphocyte, CD3+, CD4+, CD8+, NK cell counts, CD4/CD8 ratio, TNF-α , IFN- γ , I L-12p70
***Shing et al. (2007a)***	***Humans (males)***	***27 ± 2***	***69.3 ± 1.3***	***trained***	***High intensity cycle training >VT. Day 1: 20 × 1 min at PPO, 2 min recovery at 50 W; Day 2: 60 min at 100%VT; Day 3: 12 × 30 s sprints at 175% PPO, 4.5 min at 50 W recovery; Day 4: 30 mins at 80%VT, 45 min at 100%VT; Day 5: 40 min TT***	***5 days***	***Flow cytometry***	***NKCC %lysis***
Smith and Myburgh. (2006)	Humans (males)	22.6 ± 4.7	56.1 ± 4.7	trained	Cycling; 2d.wk-1 alternating sessions of (1) 80% PPO for 5 min, 1 min rest x 5-8 reps (increased by 1 bout.wk), and (2) 90% 5 kmTT speed for 5 km, 50% 5km speed for 20 min, 90% 5 km TT speed 5 km.	4 weeks	Flow cytometry	CD3+, CD4+, CD8+, NK cell counts, CD4/CD8 ratio
***Sugiura et al. (2002)***	***Rodents (males)***	***6 week***	***Not Applicable***	***Not Applicable***	***Wheel running 3 d.wk-1, 12 h.d-1. Distance increased from 7 km.d-1 to 8km.d-1, peaking at 10 km.d-1 at 5 wk.***	***8 weeks***	***CON A and PHA induced thymidine incorporation via liquid scintillation***	***Lymphocyte proliferation***
Tanimura et al. (2009)	Humans (males)	19.6 ± 0.9	46.8 ± 3.4	trained	Kendo training; 310 min.d^-1^	6 days	Flow cytometry	Lymphocyte, CD4+, CD8+ cou nts
***Verde T . J et al. (1992)***	***Humans (males)***	***288 ± 1.7***	***>60***	***trained***	***Increased running training load by via***	***3 weeks***	***Flow cytometer, PHA and CON A induced thymidine incorporation via liquid scintillation***	***CD4/CD8 ratio, lymphocyte proliferatio , resting CD3+ count***
Verde T et al. (1992)	Humans (males)	28.8 ± 1.7	65.3 ± 4.9	trained	Increased running training load by 38%	3 weeks	Flow cytometry	CD4+, CD8+ counts
Wang et al. (2011)	Humans (males)	23.1 ± 0.8	43.9 ± 2.3	untrained	Cycling at 50%Wmax, 30 min.d^-1^, 5 d.wk^-1^	4 weeks	Flow cytometry	NK cell count^+^ NK CD45RO/RA+ count
Wang et al. (2011)	Humans (males)	21.5 ± 0.7	44.1 ± 2.5	untrained	Cycling at 50% Wmax, 30 min.d^-1^, 5 d.wk^-1^	4 weeks	Flow cyt^+^metry	Lymphocyte, CD4+, CD3+, CD8+ counts, CD4/CD8 ratio
Watson (1986)	Humans (males)	22.8 ± 4.7	54.0 ± 3. d	untrained	Running 40–50 min.d^-1^, 5d.wk^-1^ at 70%–85% $\dot{V}$ O_2max_	15 weeks	Haemocytometer, Flow cytometry, NK cell ^51^Cr release	Lymphocyte and CD3+ counts, NKCC, T cell proliferation
Weng et al. (2013)	Humans (males)	22.3 ^+^0.2	46.5 ± 1.7	untrained	Cycling 5d.wk^-1^; 3 min intervals at 40 and 80% $\dot{V}$ O_2max_ , 30 min.d^-1^	5 weeks	Flow cytometry	Lymphocyte,–CD4+, CD8+ counts, CD4.CD8 ratio
Witard et al. (2012)	Humans (males)	27 ± 8	64.2 ± 6.5	trained	1 wk normal, 1 wk high intensity cycling (increase volume and intensity by 70% vs normal), 1-2 sessions.d^-1^, days7d.wk. E d of each week; 120 min at 60% $\dot{V}$ O_2ax_ and 45 min TT at 85%–100% $\dot{V}$ O_2max_	2 weeks	Flow cytometry	Lymphocyte and CD8+ counts
Zhang et al. (2019)	Humans (males)	20.1 ± 2.4	No Information	trained	High intensity training, 8 h.d^-1^ at grade 5-6 intensity	4 weeks	Flow cytometry	L–mphocyte, CD4+, C days8+ and CD3+ counts, and CD /CD8 ratio.

### 3.3 Meta-analysis

#### 3.3.1 Immune cell counts

##### 3.3.1.1 Total lymphocytes

**FIGURE 2:**
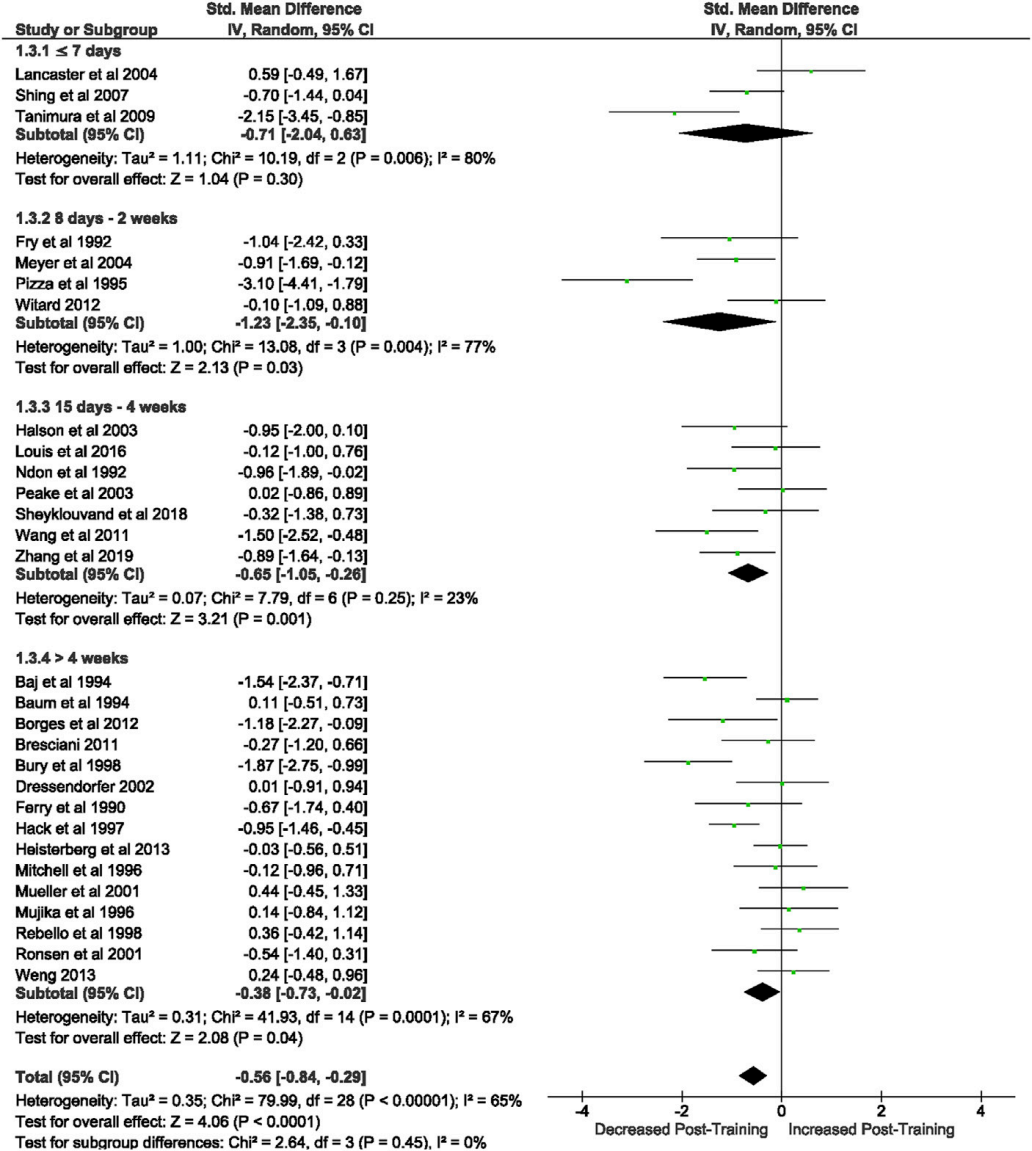
Effect of intensified training on resting lymphocyte counts, measured by FACS or Automated cell counter. Subgroup analysis is based on the duration of intensified training period; ≤ 7 days, 8 days—2 weeks, 15 days—4 weeks and > 4 weeks. All studies used human participants. *CI* Confidence interval.

Of the 57 included studies, 29 studies assessed lymphocyte count at rest. Overall, a period of intensified training significantly (Z = 4.07 (*p* < 0.0001)) reduced resting lymphocyte number with a moderate effect (*d=* −0.57, 95% CI [−0.85, −0.30]; Figure 2). However, there is substantial heterogeneity amongst the studies (Chi^2^ = 79.50, df = 28 (*p* < 0.00001), I^2^ = 65%). Subgroup analysis indicated significant decreases in resting lymphocyte count in all exercise durations of >7 days (8 days- 2 weeks (*n* = 4): Z = 2.29 (*p* = 0.02), *d =* −1.36, 95% CI [−2.53, −0.20]; 15 days- 4 weeks (*n* = 7): Z = 3.21 (*p* = 0.001), *d =* −0.65, 95% CI [−1.05, −0.26]; >4 weeks (*n* = 15): Z = 2.08 (*p* = 0.04), *d =* −0.38, 95% CI [−0.73, −0.02]). Exercise durations of ≤7 days (*n* = 3) did not alter lymphocyte counts at rest (Z = 1.04 (*p* = 0.30), *d =* −0.71, 95% CI [−2.04, −0.63]).

Of the 57 included studies, 8 studies assessed lymphocyte count in response to exercise. Overall, a period of intensified training did not change the total lymphocyte count immediately post exercise (Z = 1.47, (*p* = 0.14), *d=* −0.64, 95% CI [−1.50, 0.21]). There is substantial heterogeneity amongst the studies (Chi^2^ = 34.43, df = 7 (*p* < 0.0001), I^2^ = 80%).

###### 3.3.1.2 T cells

####### 3.3.1.2.1 CD3^+^ T cells

**FIGURE 3:**
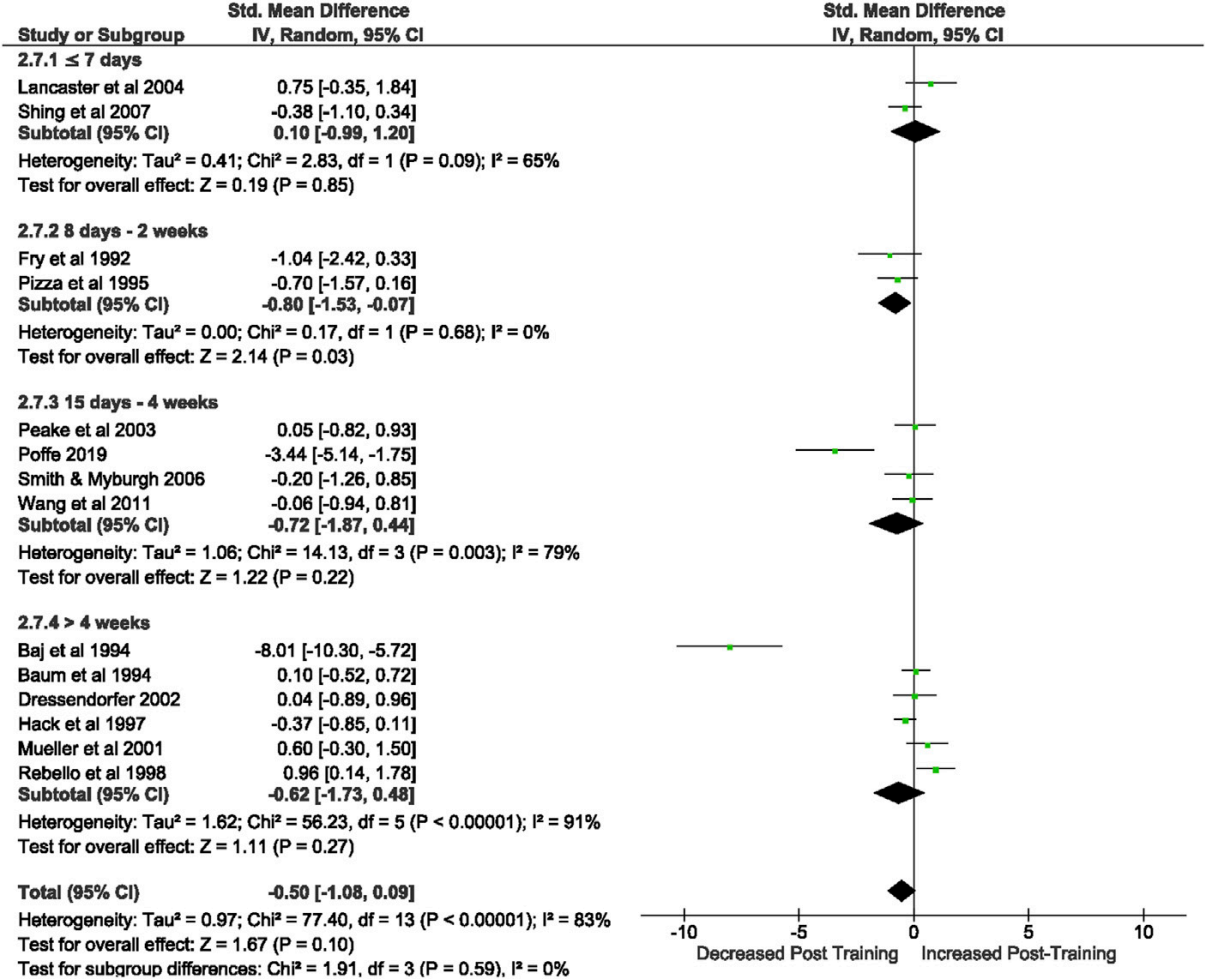
Effect of intensified training on resting CD3+ T cell counts, measured by FACS or Automated cell counter. Subgroup analysis is based on the duration of intensified training period; ≤ 7 days, 8 days—2 weeks, 1 days—4 weeks and > 4 weeks. All studies used human participants. *CI* Confidence interval.

Of the 57 included studies, 14 studies assessed CD3^+^ count at rest. Overall, a period of intensified training did not change CD3^+^ count at rest (Z = 1.67, (*p* = 0.10), *d= −*0.50, 95% CI [*−*1.08, 0.09]; Figure 3). However, there is considerable heterogeneity amongst the studies (Chi^2^ = 77.41, df = 13 (*p* < 0.00001), I^2^ = 83%). Subgroup analysis revealed that intensified training periods of 8 days- 2 weeks (n = 3) significantly decreased CD4^+^ T cell count at rest (Z = 2.14 (*p* = 0.03), *d = −*0.80, 95% CI [*−*1.53, *−*0.07]).

Of the 57 included studies, 3 studies assessed CD3^+^ count immediately post exercise. Overall, a period of intensified training did not change CD3^+^ count immediately post exercise (Z = 0.82, (*p* = 0.41), *d= −*1.16, 95% CI [*−*3.93, 1.16]). However, there is considerable heterogeneity amongst the studies (Chi^2^ = 35.40, df = 2 (*p* < 0.00001), I^2^ = 94%).

####### 3.3.1.2.2 CD4^+^ T cells

**FIGURE 4:**
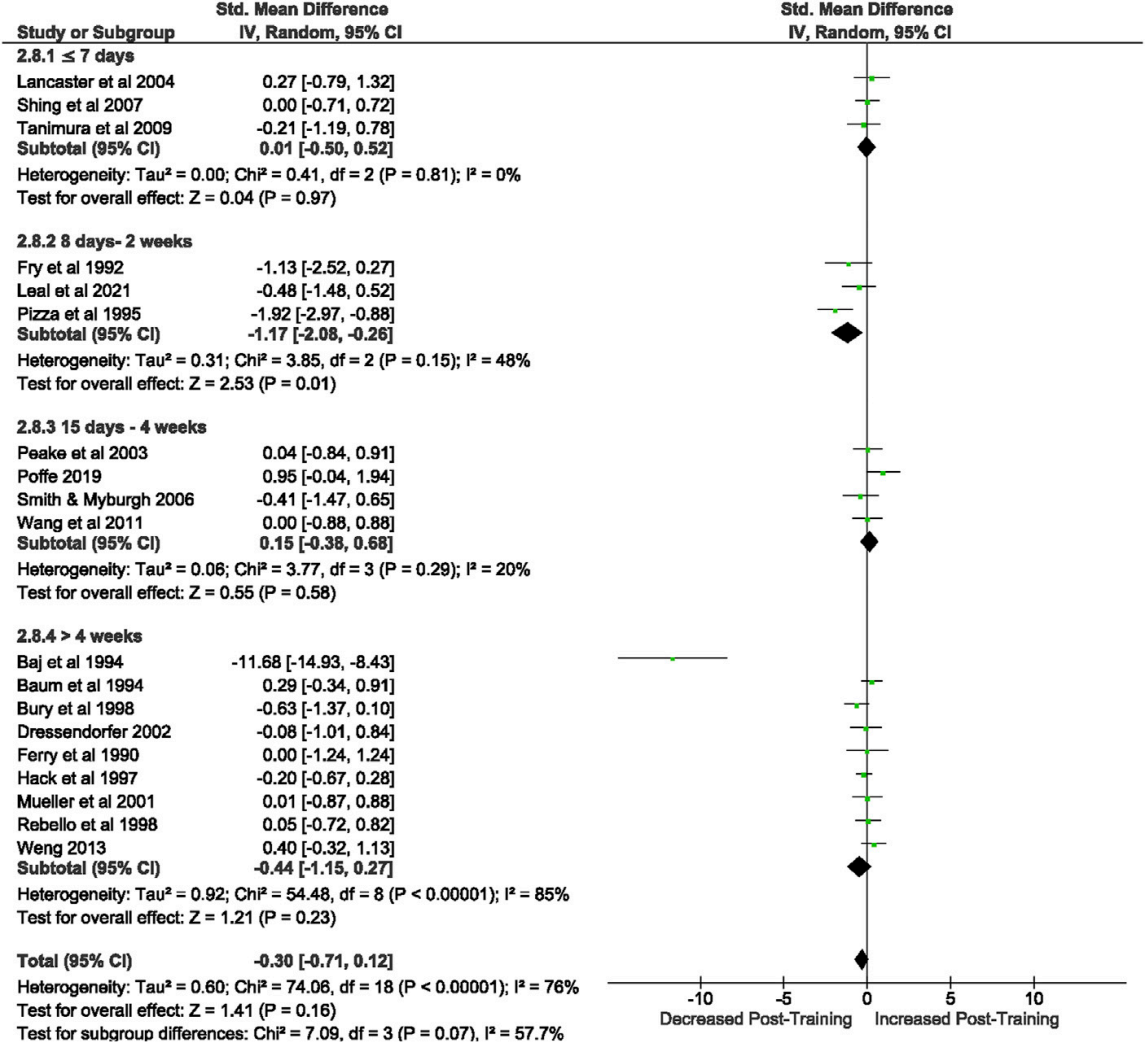
Effect of intensified training on resting CD4+ T cell counts, measured by FACS or Automated cell counter. Subgroup analysis is based on the duration of intensified training period; ≤ 7 days, 8 days—2 weeks, 15 day—4 weeks and > 4 weeks. All studies used human participants. *CI* Confidence interval.

Of the 57 included studies, 19 studies assessed CD4^+^ count at rest. Overall, a period of intensified training did not change CD4^+^ count at rest (Z = 1.41, (*p* = 0.16), *d= −*0.30, 95% CI [*−*0.71, 0.12]; Figure 4). However, there is considerable heterogeneity amongst the studies (Chi^2^ = 74.12, df = 18 (*p* < 0.00001), I^2^ = 76%). Subgroup analysis revealed that intensified training periods of 8 days- 2 weeks (*n* = 3) significantly decreased CD4^+^ T cell count at rest (Z = 2.53 (*p* = 0.01), *d = −*1.17, 95% CI [*−*2.08, *−*0.26]).

Of the 57 included studies, 5 studies assessed CD4^+^ count immediately post exercise. Overall, a period of intensified training did not change CD4^+^ count immediately post exercise (Z = 0.80, (*p* = 0.42), *d =* 0.35, 95% CI [*−*0.50, 1.19]). However, there is substantial heterogeneity amongst the studies (Chi^2^ = 12.92, df = 4 (*p* = 0.01), I^2^ = 69%).

####### 3.3.1.2.3 CD8^+^ T cells

**FIGURE 5:**
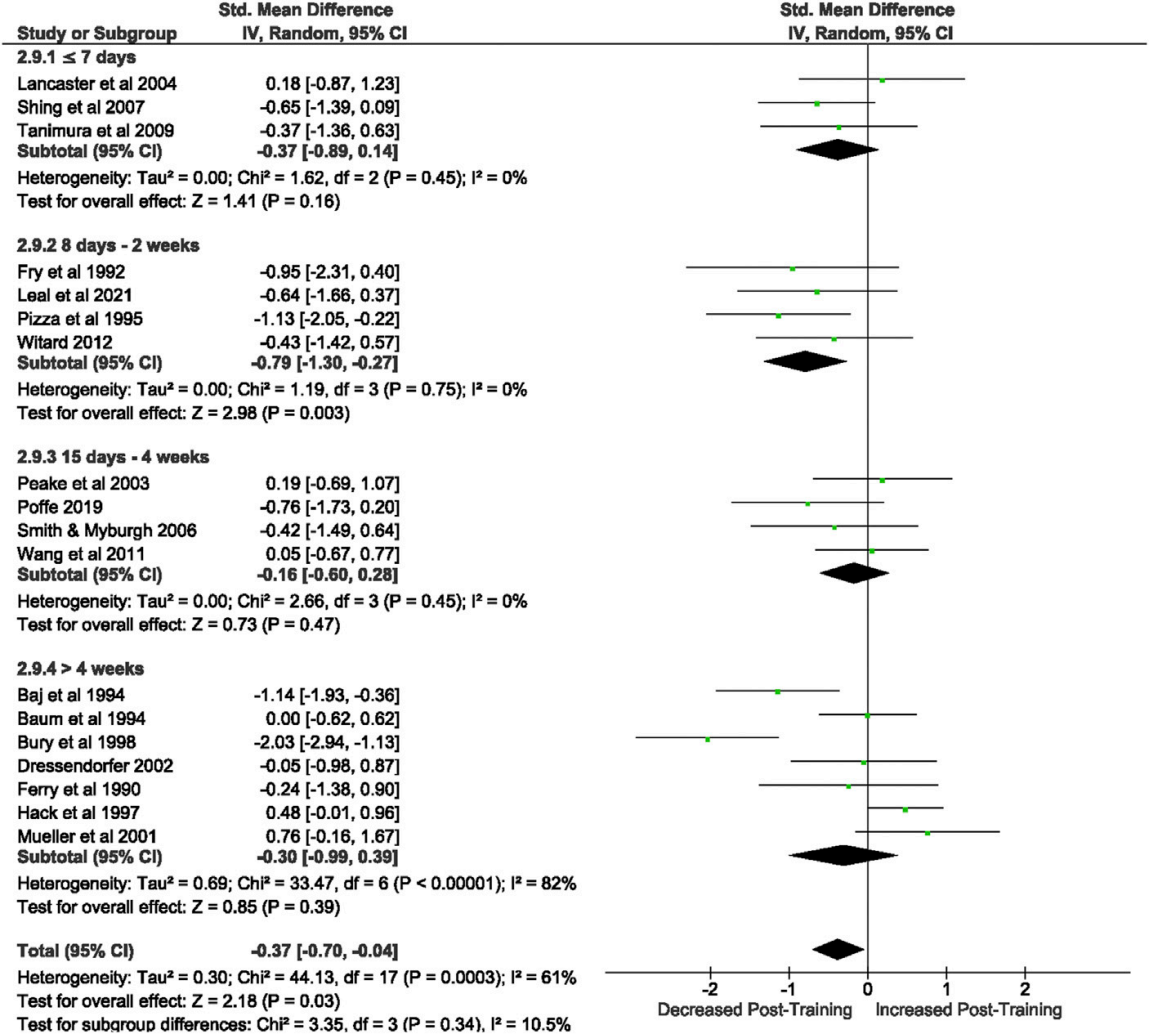
Effect of intensified training on resting CD8+ T cell counts, measured by FACS or Automated cell counter. Subgroup analysis is based on the duration of intensified training period; ≤ 7 days, 8 days—2 weeks, 15 day—4 weeks and > 4 weeks. All studies used human participants. *CI* Confidence interval.

Of the 57 included studies, 18 studies assessed CD8^+^ count at rest. Overall, a period of intensified training significantly [Z = 2.18, (*p* = 0.03)] reduced CD8^+^ count at rest with a small effect (*d= −*0.37, 95% CI [*−*0.7, *−*0.04]; Figure 5). However, there is substantial heterogeneity amongst the studies (Chi^2^ = 44.14, df = 17 (*p* = 0.0003), I^2^ = 61%). Subgroup analysis revealed that intensified training periods of 8 days- 2 weeks (*n* = 4) were the only duration to significantly alter resting CD8^+^ T cell count (Z = 2.98 (*p* = 0.003), *d = −*0.79, 95% CI [*−*1.30, *−*0.27]).

Of the 57 included studies, 6 studies assessed CD8^+^ count immediately post exercise, before and after a period of overtraining. Overall, a period of intensified training did not change CD8^+^ count immediately post exercise (Z = 0.54, (*p* = 0.59), *d =* 0.37, 95% CI [*−*0.97, 1.72]). However, there is considerable heterogeneity amongst the studies (Chi^2^ = 45.96, df = 6 (*p* < 0.00001), I^2^ = 89%).

####### 3.3.1.2.4 CD4/CD8 Ratio

**FIGURE 6:**
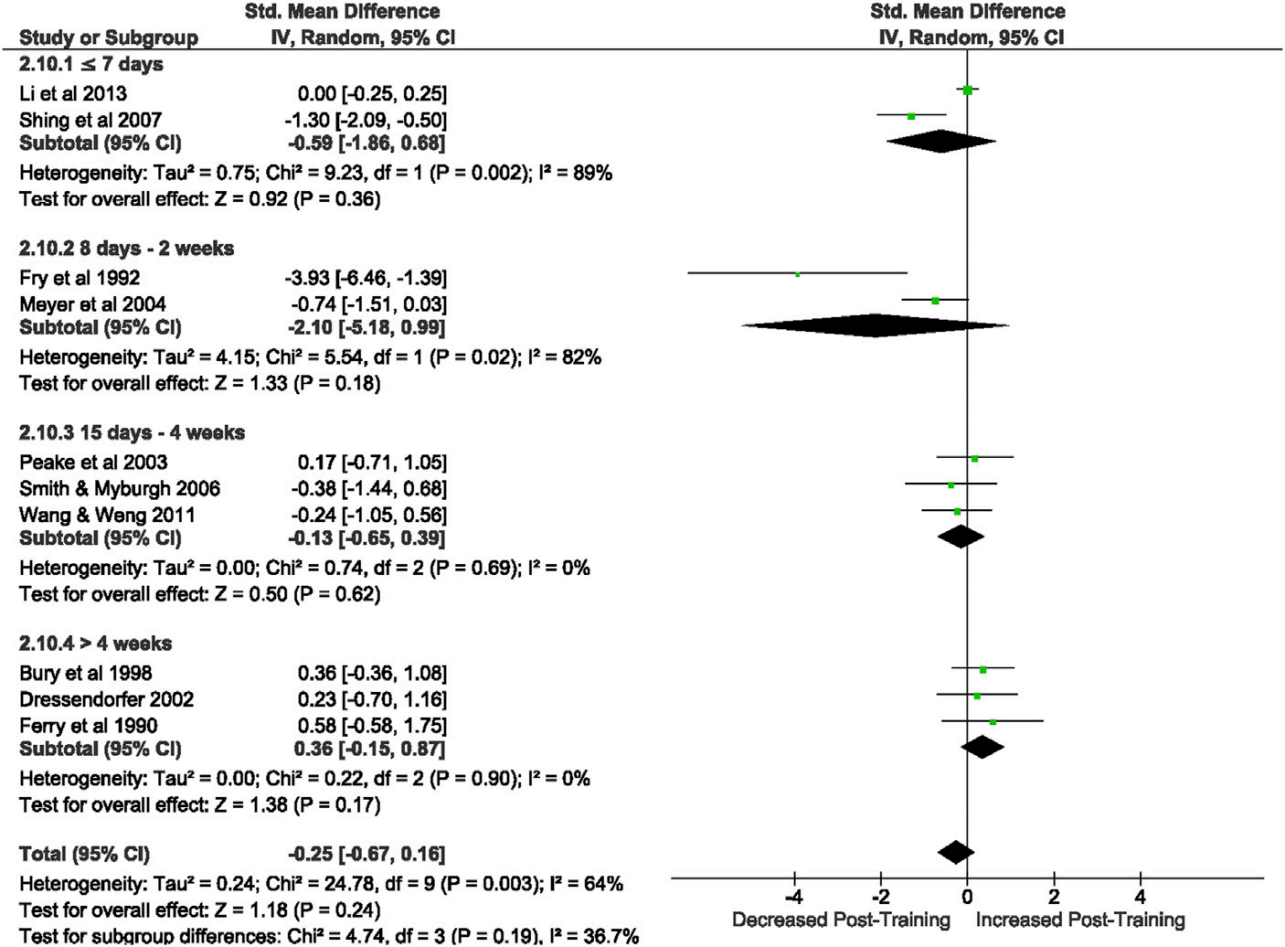
Effect of intensified training on resting CD4/CD8 ratio. Subgroup analysis is based on the duration of intensified training period; ≤ 7 days, 8 days—2 weeks, 15 days—4 weeks and > 4 weeks. Studies used a mixtureof human and rodent (Blank et al., 1994; Kaufman et al., 1994) participants. *CI* Confidence interval.

Of the 57 included studies, 19 studies assessed CD+/CD8+ Ratio at rest. Overall, a period of intensified training did not change the resting CD4+/CD8+ ratio (Z = 0.91, (*p* = 0.36), *d= −*0.15, 95% CI [-0.49, 0.18]; Figure 6). However, there is substantial heterogeneity amongst the studies (Chi^2^ = 59.81, df = 18 (*p* < 0.00001), I^2^ = 70%).

Of the 57 included studies, 7 studies assessed the CD4+/CD8+ ratio immediately post exercise. Overall, a period of intensified training did not change the CD4+/CD8+ ratio immediately post exercise (Z = 0.19, (*p* = 0.85), *d= -*0.04, 95% CI [*−*0.43, 0.35]). There is low heterogeneity amongst the studies (Chi^2^ = 7.15, df = 6 (*p* = 0.31), I^2^ = 16%).

####### 3.3.1.2.5 Natural killer cells

**FIGURE 7:**
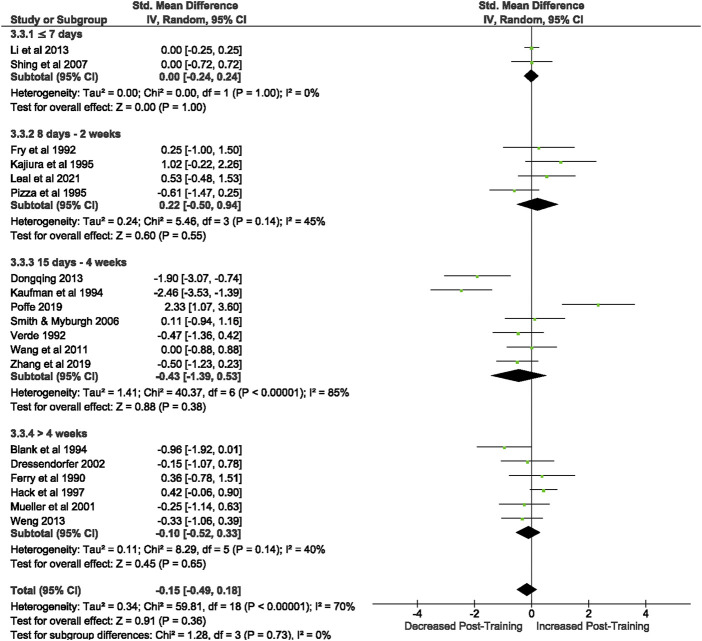
Effect of intensified training on resting Natural Killer cells, measured by FACS or Automated cell counter. Subgroup analysis is based on the duration of intensified training period; ≤ 7 days, 8 days—2 weeks, 15 days—4 weeks and > 4 weeks. All studies used human participants. *CI* Confidence interval.

Of the 57 included studies, 10 studies assessed NK cell count at rest based on CD56^+^ expression. Overall, a period of intensified training did not change NK cell count at rest (Z = 1.18, (*p* = 0.24), *d= −*0.25, 95% CI [*−*0.67, 0.16]; Figure 7). However, there is substantial heterogeneity amongst the studies (Chi^2^ = 24.78, df = 10 (*p* = 0.003), I^2^ = 64%). Subgroup analysis revealed that resting NK cell count did not alter after a period of intensified training of any duration [i.e., ≤7 days (*p* = 0.36), 8 days-2 weeks (*p* = 0.18), 15 days-4 weeks (*p* = 0.62) or >4 weeks (*p* = 0.17)].

Of the 57 included studies, 3 studies assessed NK cell count based on CD56^+^ expression immediately post exercise. Overall, a period of intensified training did not change NK cell count immediately post exercise (Z = 0.10, (*p* = 0.92), *d= 0.04*, 95% CI [−0.74, 0.82]). However, there is substantial heterogeneity amongst the studies (Chi^2^ = 4.35, df = 3 (*p* = 0.11), I^2^ = 54%).

#### 3.3.2 Immune function

##### 3.3.2.1 Lymphocyte proliferation

###### 3.3.2.1.1 Resting stimulated human peripheral blood (counts per minute)

Of the 57 included studies, 5 different studies assessed human peripheral blood lymphocyte proliferation at rest, with 3 studies assessing lymphocyte proliferation to more than one stimulant, thus 8 results were entered into the meta-analysis. Overall, a period of intensified training did not change resting lymphocyte proliferation (Z = 0.04, (*p* = 0.97), *d=* −0.02, 95% CI [−1.10, 1.05]). However, there is considerable heterogeneity amongst the studies (Chi^2^ = 75.72, df = 7 (*p* < 0.00001), I^2^ = 91%).

##### 3.3.2.2 Resting stimulated rodent spleen lymphocytes (counts per minute)

Of the 57 included studies, 6 studies assessed rodent spleen lymphocyte proliferation at rest with 2 studies assessing lymphocyte proliferation to more than one stimulant, thus 8 results were entered into the meta-analysis. Overall, a period of intensified training did not change resting spleen lymphocyte proliferation (Z = 0.10, (*p* = 0.92), *d =* 0.09, 95% CI [−1.79, 1.97]). However, there is considerable heterogeneity amongst the studies (Chi^2^ = 94.88, df = 7 (*p* < 0.00001), I^2^ = 93%).

##### 3.3.2.3 Exercise induced CON a stimulated rodent spleen lymphocytes (counts per minute)

Of the 57 included studies, 3 studies assessed spleen lymphocyte proliferation immediately post exercise. Overall, a period of intensified training did not change spleen lymphocyte proliferation immediately post exercise (Z = 0.77, (*p* = 0.44), *d=* −2.38, 95% CI [−8.44, 3.68]). However, there is considerable heterogeneity amongst the studies (Chi^2^ = 44.46, df = 2 (*p* < 0.00001), I^2^ = 96%).

#### 3.3.3 NK cell cytolytic activity

Of the 57 included studies, 3 studies assessed NK cytolytic activity as %lysis of K-562 tumour cells. Overall, a period of intensified training did not change NK cell cytolytic activity (Z = 1.63, (*p* = 0.10), *d =* 1.13, 95% CI [−0.23, 2.49]). However, there is considerable heterogeneity amongst the studies (Chi^2^ = 16.75, df = 2 (*p* < 0.0002), I^2^ = 88%).

##### 3.3.3.1 Cytokines

###### 3.3.3.1.1 Unstimulated TNF- α

**FIGURE 8:**
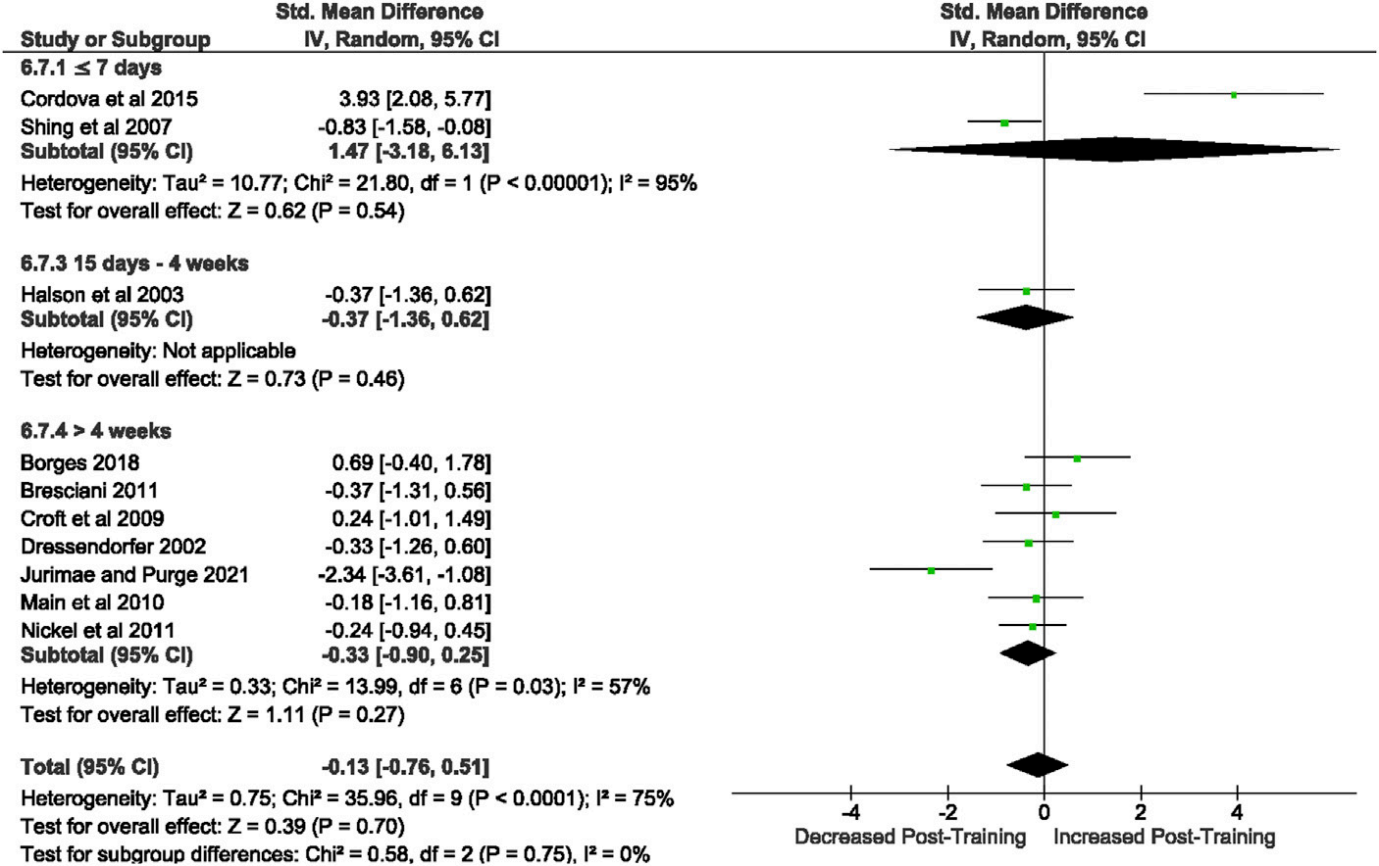
Effect of intensified training on resting unstimulated plasma TNF- α. Subgroup analysis is based on the duration of intensified training period; ≤ 7 day, 8 days—2 weeks, 15 days—4 weeks and > 4 weeks. All studies used human participants. *CI* Confidence interval.

Of the 57 included studies, 10 studies assessed unstimulated TNF-α secretion at rest. Overall, a period of intensified training did not change resting TNF- α secretion (Z = 0.39, (*p* = 0.70), *d=* −0.13, 95% CI [−0.76, 0.51]; Figure 8). However, there is substantial heterogeneity amongst the studies (Chi^2^ = 35.96, df = 9 (*p* < 0.00001), I^2^ = 75%).

####### 3.3.3.1.2 Unstimulated IFN-γ

Of the 57 included studies, 5 studies assessed unstimulated IFN-γ secretion at rest. Overall, a period of intensified training did not change resting IFN- γ secretion (Z = 1.33, (*p* = 0.18), *d =* 0.70, 95% CI [−0.33, 1.74]). However, there is considerable heterogeneity amongst the studies (Chi^2^ = 19.42, df = 4 (*p* = 0.0006), I^2^ = 79%).

####### 3.3.3.1.3 Unstimulated IL-1β

**FIGURE 9:**
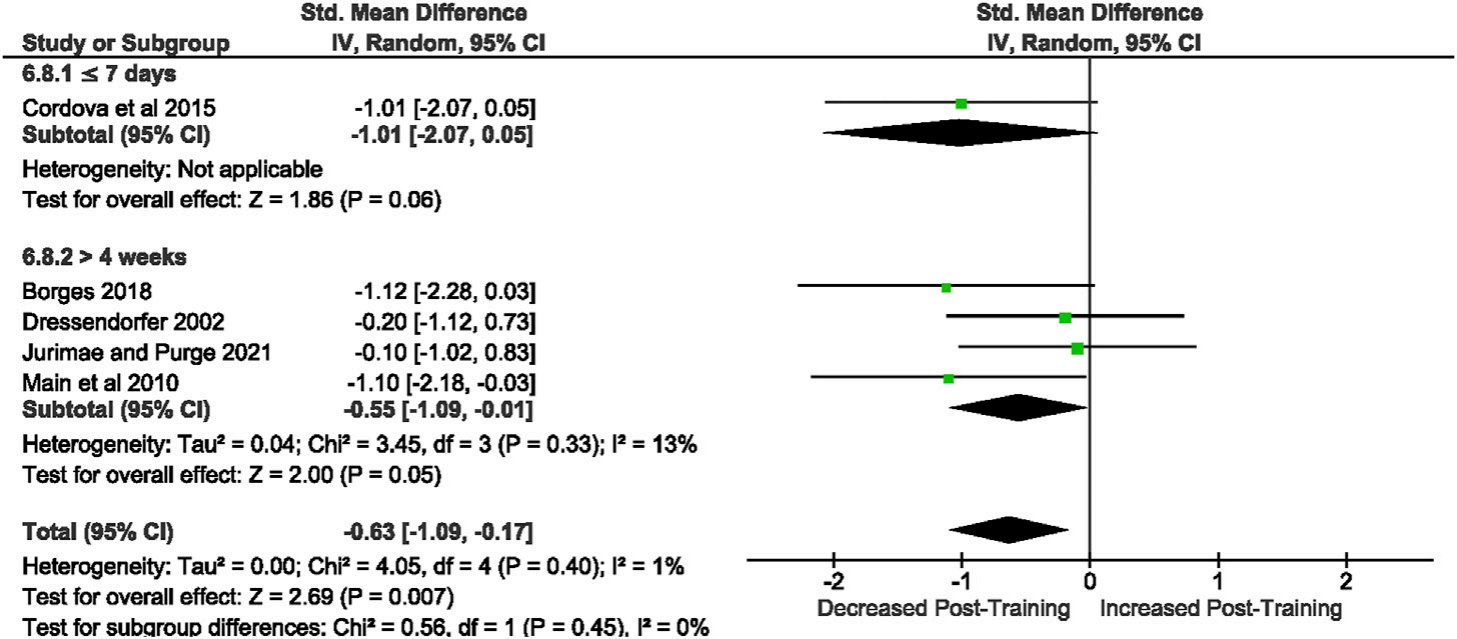
Effect of intensified training on resting unstimulated plasma IL-1 β. Subgroup analysis is based on the duration of intensified training period; ≤ 7 days, 8 days—2 weeks, 15 days—4 weeks and > 4 weeks. All studis used human participants. *CI* Confidence interval.

Of the 57 included studies, 5 studies assessed unstimulated IL-1β secretion at rest. Overall, a period of intensified training significantly [Z = 2.69, (*p* = 0.007)] decreased resting IL-1 β secretion with a moderate effect (*d=* −0.63, 95% CI [−1.09, −0.17]; Figure 9). There is very low heterogeneity amongst the studies [Chi^2^ = 4.05, df = 4 (*p* = 0.40), I^2^ = 1%].

####### 3.3.3.1.4 Unstimulated IL-10

Of the 57 included studies, 3 studies assessed unstimulated IL-10 secretion at rest. Overall, a period of intensified training did not change resting IL-10 (Z = 1.62, (*p* = 0.11), *d= 1.52*, 95% CI [−0.32, 3.37]). However, there is considerable heterogeneity amongst the studies [Chi^2^ = 12.59, df = 2 (*p* = 0.002), I^2^ = 84%].

####### 3.3.3.1.5 Unstimulated IL-2

Of the 57 included studies, 2 studies assessed unstimulated IL-2 secretion at rest. Overall, a period of intensified training did not change resting IL-2 secretion (Z = 1.62, (*p* = 0.11), *d= 1.41*, 95% CI [−0.30, 3.12]). However, there is considerable heterogeneity amongst the studies [Chi^2^ = 4.26, df = 1 (*p* = 0.04), I^2^ = 77%].

###### 3.3.3.2 Dendritic cells (all rodent studies)

####### 3.3.3.2.1 Dendritic cell CD80 expression

**FIGURE 10:**
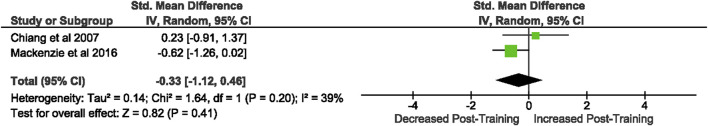
Effect of intensified training on resting dendritic cell CD80 expression. All studies used rodent participants. *CI* Confidence interval.

Of the 57 included studies, 2 studies assessed DC CD80 expression as a percentage of fluorescent intensity. Overall, a period of intensified training did not change rodent bone marrow derived DC CD80 expression (Z = 0.82, (*p* = 0.41), *d =* −0.33, 95% CI [−1.12, 0.46]; Figure 10). There is low heterogeneity amongst the studies [Chi^2^ = 1.64, df = 1 (*p* = 0.20), I^2^ = 39%].

####### 3.3.3.2.2 Dendritic cell MHC II expression

**FIGURE 11:**
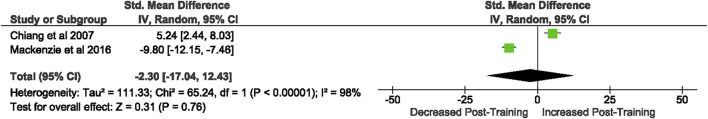
Effect of intensified training on resting dendritic cell MHC II expession. All studies used rodent participants. *CI* Confidence interval.

Of the 57 included studies, 2 studies assessed DC MHC II expression. Overall, a period of intensified training did not change rodent DC MHC II expression (Z = 0.31, (*p* = 0.76), *d=* −2.30, 95% CI [−17.04, 12.43]; Figure 11). However, there is considerable heterogeneity amongst the studies [Chi^2^ = 65.24, df = 1 (*p* < 0.00001), I^2^ = 98%].

####### 3.3.3.2.3 DC CD86 expression

**FIGURE 12:**
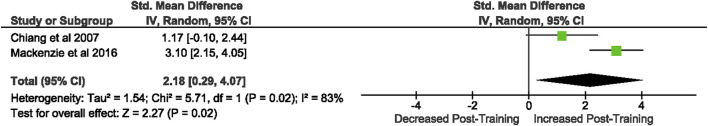
Effect of intensified training on resting dendritic cell CD86 expression. All studies used rodent participants. *CI* Confidence interval.

Of the 57 included studies, 2 studies assessed rodent DC CD86 expression as a percentage of fluorescent intensity. Overall, a period of intensified training significantly [Z = 2.99, (*p* = 0.003)] increased DC CD86 expression with a large effect (*d =* 2.18, 95% CI [0.29, 4.07]; Figure 12). However, there is substantial heterogeneity amongst the studies [Chi^2^ = 3.60, df = 1 (*p* = 0.06), I^2^ = 72%].

## 4 Discussion

### 4.1 Overview

The purpose of this study was to bring together a body of research to characterise the T lymphocyte, NK cell and DC activity at rest and in response to exercise stress following periods of intensified training. To assess whether these immune biomarkers could provide insight into their use as diagnostic indicators of the negative states of overtraining, it was necessary to focus on research with appropriate intensity of training. Training protocols of >7 days are more likely to induce NFOR than shorter training periods of the same intensity (Halson et al., 2002). With 93% of included studies utilising a training protocol of >7 days in duration, we can be confident that NFOR was a possibility. In agreement with this, subgroup analysis conducted on resting immune cell counts within this review indicated that training durations of <7 days did not induce alterations in immune cell counts, whereas durations above this did. Understanding the relationship between resting and exercise induced immune cell markers and intensified training periods may be useful in the diagnosis of the negative states of overtraining e.g., NFOR or OTS. Currently no clear biomarker has been uncovered to support diagnosis of these states (Meeusen et al., 2013). Additionally, this review highlights further some gaps within the literature where further experimental research is required.

In total this meta-analysis examined 16 immune biomarkers; 7 were assessed at rest and in response to a bout of exercise, with the remaining 9 assessed at rest only. When comparing each variable from before to after an intensified training period, significant decreases in resting total lymphocyte and CD8^+^ T cell counts, and unstimulated plasma IL-1β levels were found. In addition, resting DC CD86 expression was significantly increased in rodents only.

### 4.2 Total lymphocyte and CD8^+^ T cell counts at rest

The meta-analysis found a significant decrease in resting total lymphocyte and CD8^+^ T cell count after a period of intensified training. The magnitude, direction, and length of immune recovery after a period of intensified training is dependent upon the intensity, duration, and load of training (Simpson et al., 2020). Therefore, differences in exercise protocols used by studies utilised in this review may account for the inter-study variability in findings. To examine this further, we note that of the studies reporting intensity increases, the study displaying the largest effect size for a significant decrease in resting lymphocyte count elevated running training load by 200% across a 10-day period in trained participants (Pizza et al., 1995). Whereas the study with the lowest effect size increased cycling training load by 40% across a 13-day period in trained participants (Meyer et al., 2004). The use of sudden increases in workloads as large as 200% may not be reflective of practice within elite sport which could be suggested to limit the relevance of the findings to the wider athletic population. However, an overload of the immune system does provide insight into its response to training stress and helps highlight whether immune biomarkers could act as diagnostic indicators of NFOR or OTS. Although this may not be reflective of best practice in an athletes every-day regime, some individuals may experience this overload. Additionally, the heart rate response and number of active muscles during running is known to be considerably higher than that of cycling exercise. In triathletes, it has been observed that the maximum heart rate achieved during cycling is often 6–10 bpm lower than obtained during running (Millet et al., 2009). Therefore, aside from the obvious intensity differences between Pizza et al. (1995) and Meyer et al. (2004), the use of running exercise in Pizza et al. (1995) induces larger internal stress and is thus more likely to push an athlete towards a state of NFOR than cycling protocols for the same given intensity and/or duration.

During exercise a transient period of lymphocytosis occurs followed by a period of lymphocytopenia after cessation of exercise (Shek et al., 1995). Traditionally it is thought that this lymphocytopenia creates a 3–72 h window of opportunity for infection (Pedersen and Ullum, 1994) which is prolonged and more severe if a second bout of exercise is performed within this time frame (Simpson et al., 2015). Exercise-induced lymphocyte apoptosis (Phaneuf & Leeuwenburgh, 2001) that can still be evident 24 h after a single bout of treadmill exercise to exhaustion (Mars et al., 1998) has been suggested as a possible cause for this. This mechanism is thought to be mediated by cortisol (Riccardi et al., 1999) by binding to glucocorticoid receptors within immune cells, leading to increased cell apoptosis (Cain and Cidlowski, 2017). A single bout of exercise (<1.5 h) has been shown to increase reactive oxygen species (ROS) and is further increased with higher intensities (Thirupathi et al., 2021), often peaking 2–3 days after exercise (Theodorou et al., 2011). This increase in ROS has also been attributed to initiating lymphocyte apoptosis *via* damaging the DNA of the immune cell (Mooren et al., 2004). Reactive oxygen species are oxygen containing molecules that are capable of independent existence, containing at least one or more unpaired electrons (Jakubczyk et al., 2020). At low levels, ROS may function in cell signalling processes, regulating cell growth and differentiation, inflammation, immune responses and immune survival (Romero and Agostinis, 2014). However, at higher levels, ROS may damage cellular DNA of immune cells and thus play a role in apoptosis (Mooren et al., 2004).

As the studies used in this review examined repeated exposures to exercise stress, exceeding the aforementioned duration and/or intensities, apoptosis could be a potential reason for the decrease in resting lymphocyte numbers found after a period of intensified training. Despite earlier investigations suggesting that post-exercise lymphocytopenia is a result of apoptosis, Simpson et al. (2007) reported limited lymphocyte markers of apoptosis (Annexin-V (+) or HSPA60) 1 h after treadmill exercise to fatigue completed at 80% $\dot{V}$ O_2max._, yet lymphocytopenia was evident. Moreover, the levels of apoptosis reported in studies indicating an increase in cell death are usually very small i.e., <5% (Mooren et al., 2002; Simpson et al., 2007), and as such is unlikely to account for the 30%–40% reductions in blood lymphocyte count witnessed after exercise (Peake et al., 2016). Therefore, the reduced resting lymphocyte and CD8^+^ T cell counts after a period of intensified training could instead be due to an exercise-driven redistribution of highly functional effector cells, such as CD8^+^ T cells from the blood stream into the tissues and organs for heightened identification and eradication of tissue tumour cells (Simpson et al., 2020).

This redistribution has been demonstrated in rodents *via* fluorescent cell tracking following both running and swimming exercise (Kruger et al., 2007). In humans, cycling at 85% of maximum power output (Wmax) for 20 min prompted the preferential mobilisation of highly cytotoxic CD8^+^ T cells possessing a high propensity to migrate into the peripheral tissues during exercise recovery (Campbell et al., 2009). The redistribution of highly functional effector cells is driven by increased haemodynamics and the release of catecholamines and glucocorticoids following the activation of the sympathetic nervous system and HPA axis (Simpson et al., 2015).

Catecholamines, for example adrenaline and noradrenaline, influence the mobilisation of CD8^+^ T cells both directly, *via* the action of adrenaline on lymphocyte β_2_-adrenergic receptors (Graff et al., 2018) and expression of adhesion molecules (Shephard, 2003), and indirectly, *via* increased cardiac output and shear stress mobilising lymphocytes from endothelial walls (Shephard, 2003). Both mechanisms result in the demargination of highly cytotoxic effector cells into the circulation (Dimitrov et al., 2010). CD8^+^ cells are the T cell subset expressing the most adrenergic receptors (β_2_ receptor) and are therefore more susceptible to change with increased exposure to catecholamines across the training period (Shephard, 2003). Whilst catecholamines drive the lymphocytosis of CD8^+^ T cells during exercise, glucocorticoids such as cortisol are thought to influence the egress of CD8^+^ T cells out of the peripheral blood and into the peripheral tissues and organs during exercise recovery. This is believed to be *via* heightened expressions of certain cell activation and adhesion molecules that facilitate migration, enabling them to pass through endothelial cells and into tissues (Simpson et al., 2006). During prolonged recovery from intensified exercise, substantial infiltration of certain subsets of T lymphocytes into damaged skeletal muscles also occur in order to enhance muscle repair (Jones et al., 1986). It is therefore possible that the reduction in resting lymphocytes, and more specifically CD8^+^ T cells, is the result of a redistribution into damaged muscles, caused by repeated bouts of exercise over the training period (Pizza et al., 1995).

### 4.3 Exercise induced lymphocyte counts

Despite significant decreases in resting lymphocyte counts after a period of intensified training, there was no overall significant change in lymphocyte counts in response to an acute bout of exercise. This suggests that the lymphocyte response to exercise stress remains unchanged both before and after a period of intensified training. 8 studies investigated the exercise induced changes in lymphocyte count after a period of intensified training. Of those 8 studies, only 1 found a significant increase in exercise induced lymphocyte count (Wang et al., 2011). All other studies included either found no change (Ferry et al., 1990; Ndon et al., 1992; Ronsen et al., 2001; Witard et al., 2012) or a significant decrease (Lancaster et al., 2004; Shing et al., 2007; Hasanli et al., 2021).

On examination of the acute exercise bouts used before and after the intensified training period to assess the exercise induced lymphocyte changes in each study; all were all-out tests until volitional exhaustion. Differences in exercise intensities of these acute exercise bouts are therefore not the cause of the differences in lymphocyte response between Wang et al. (2011) and the rest of the studies. However, Wang et al. (2011) was the only study that used untrained, sedentary participants, with an average $\dot{V}$ O_2max_ of 44.1 ml/kg/min (classed as “Fair” (ACSM, 2017). All of the other studies utilised participants with a $\dot{V}$ O_2max_ > 60 ml/kg/min; classed as “Superior” (ACSM, 2017). It is known that trained and untrained individuals undergoing a period of intensified training show different cellular responses to exercise, thought to be related to differences in the elevated cortisol levels and alterations in the pro/anti-inflammatory balance in response to exercise (Walsh et al., 2011). Specifically, T cell counts appear to be sensitive to exercise load in well trained individuals undertaking a period of intensified training, but this sensitivity is reduced in sedentary individuals undertaking the same training (Walsh et al., 2011). If Wang et al. (2011) was to be removed, and the meta-analysis re-run using only trained participants, a significant reduction in total lymphocyte counts in response to an acute bout of exercise would be found.

Additionally, the intensity of the training intervention used by Wang et al. (2011) was lower than those used by the other studies assessing exercise induced lymphocyte counts. Wang et al. (2011) used a training intensity of 50% W_max_ which has previously been likened to ∼55% $\dot{V}$ O_2max_ (Van Loon et al., 1999). According to Gore et al. (2013), any exercise <60% $\dot{V}$ O_2max_ is classed as light aerobic and represents the lowest training zone. The two studies displaying significant increases in exercise induced lymphocyte counts utilised intensities above the ventilatory threshold level (Shing et al., 2007) and 70–95% HRmax for most of the training duration (Lancaster et al., 2004). It is suggested that anaerobic exercise during maximal effort is the most powerful catecholamine and cortisol stimulator (Baj et al., 1994), therefore the differences in responses between these studies could be because the training protocol adopted by Wang et al. (2011) was not intense enough to elicit such hormonal changes that may blunt the exercise induced lymphocytosis post training that was seen in Lancaster et al. (2004) and Shing et al. (2007).

### 4.4 Resting unstimulated IL-1β

A significant decrease in resting unstimulated plasma IL-1β levels after a period of intensified training was found. High serum levels of IL-1β are thought to exacerbate damage during chronic disease and acute tissue injuries (Lopez-Castejon and Brough, 2011); commonly implicated in the pathogenesis of chronic diseases such as Rheumatoid Arthritis (Altomonte et al., 1992), Atherosclerosis (Kirii et al., 2003) and Chronic Obstructive Pulmonary Disease (Hammad et al., 2015). As such, a reduction in resting serum IL-1β has been implicated in reducing low grade inflammation and is a target for many anti-inflammatory treatments (Dinarello et al., 2012). This indicates that reduced resting IL-1β levels after a period of training may be seen as a positive anti-inflammatory effect of exercise training. Conversely, IL-1β is essential for resistance to infections, and lower resting levels of IL-1β may reduce the ability to initiate a Type 1 immune response (Murray, 2013). However, in order to understand the true effects this reduction may have on immunity, stimulated cytokine release needs to be assessed.

On examination of the individual studies there needs to be a consideration of how resting IL-1β was defined. Córdova Martinez et al., 2015 took their post training, resting sample 3 h after a cycling race and Main et al. (2010) collected what they referred to as a resting sample 30 min after a water-based rowing session. A study investigating plasma and mononuclear mRNA IL-1β levels in response to a 3-h mixed cycling and running bout at 60%–65% $\dot{V}$ O_2max_ reported that plasma IL-1β levels were still elevated compared to resting pre-exercise levels at both 300 min and 24 h after cessation of exercise, but no change in mRNA was detected (Moldoveanu et al., 2000). Similarly, an acute bout of plyometric exercises consisting of 50 jumps and 50 drop jumps (Chatzinikolaou et al., 2010), and a marathon race (Ostrowski et al., 1999) have been shown to elevate plasma IL-1β levels immediately after exercise. It could therefore be argued that the true resting plasma IL-1β responses have not been shown and may account for the large differences in effect sizes between these studies and the two studies showing the smallest effect sizes. The two studies showing the smallest effect sizes, indicating little to no change in resting IL-1β levels, collected blood samples after at least 24 h of rest (Dressendorfer et al., 2002; Jurimae and Purge, 2021).

In both of the studies displaying little to no change in IL-1β, markers of performance were shown to increase. For example, in a group of competitive endurance cyclists, after 6.5 weeks of intensified training, average heart rate decreased at submaximal levels, cycling economy improved and no change in the testosterone:cortisol ratio was observed that would indicate any physiological stress that may evoke immune changes (Dressendorfer et al., 2002). Likewise, a group of elite rowers undergoing 6 months of volume extended training saw an improvement in performance in the form of increased aerobic power, indicating normal training adaptation (Jurimae and Purge, 2021). Therefore, regardless of sample timing, the results of these two studies may not be representative of the overtrained athlete, and insight into their use as a biomarker of overtraining may be limited.

Although acute bouts of exercise have been shown to elevate unstimulated plasma IL-1β, this review found that a period of intensified training led to significantly reduced levels. Pro-inflammatory cytokines are mediated by both anti-inflammatory cytokines, such as IL-1ra and IL-6, and cytokine inhibitors, such as cortisol and adrenaline, which are known to increase markedly in the circulation following endurance exercise (Suzuki et al., 2002). Cortisol is known to possess anti-inflammatory effects (Blannin et al., 1996; Ortega et al., 1996) and adrenaline has been shown to downregulate the stimulated production of IL- 1β (Bergmann et al., 1999). Additionally, IL-6, the most notable cytokine secreted from contracting muscles, increases up to 100-fold during exercise, resulting in increased anti-inflammatory cytokine production, and decreased IL-1β production (Beavers et al., 2010). As such, the triggered anti-inflammatory effects of exercise could explain the significant decrease in resting IL-1β levels found after a period of intensified training in this review.

Whilst this review focused on unstimulated IL-1β concentrations, it is apparent that stimulated cytokine production from immune cells may be more informative of the overall immune state (Gleeson et al., 2013, P. 299). This is because IL-1β does not increase exponentially during exercise, which is different when compared to infections (Pedersen and Hoffman-Goetz, 2000). Therefore, stimulating the cytokine response from immune cells after exercise with stimulants such as LPS, mimics the initial innate immune response to bacterial infection. In line with this, Nielsen et al. (2016) found no significant changes in unstimulated IL-1β immediately after a half-marathon, but when measuring LPS-stimulated cytokines after the same bout of exercise, a significant decrease in plasma IL-1β was found. Despite this, only unstimulated cytokine responses were included in the meta-analysis because limited papers using the same stimulants were available for grouping.

### 4.5 Dendritic cell markers

The meta-analysis revealed a significant upregulation of stimulated CD86 expression after a period of intensified training in rodents, yet no significant changes were found for CD80 or MHC II expression. Only two papers satisfied the search criteria for this analysis, and both were in rodents. As such, it is reasonable to suggest that strong conclusions can not be drawn.

When looking at the trends across all papers that assessed DC markers, including those not suitable for meta-analyses, it is apparent that overall, there is a trend for increased CD86 (Chiang et al., 2007; Mackenzie et al., 2016; Fernandes et al., 2019) expression after a period of training, with conflicting results for MHC II (Chiang et al., 2007; Mackenzie et al., 2016) and CD80 (Liao et al., 2006; Chiang et al., 2007; Mackenzie et al., 2016; Fernandes et al., 2019). Chiang et al. (2007) found a significant increase in DC MHC II expression and IL-12 secretion in male sprawly rats in response to 5 weeks progressive endurance treadmill running, but no significant increase in CD80/86 expression. They suggested the upregulation in MHC II and IL-12 indicates enhanced DC differentiation and maturation, potentially implicating greater antigen presentation ability, and a greater Th1 response to elicit antitumor immunity (Chiang et al., 2007). Chiang et al. (2007) utilised a periodised endurance protocol; a well-designed training programme consisting of progressive intensity increases and sufficient active recovery periods. Periodised endurance training has been shown to modulate immunity in human models (Liao et al., 2006), allowing for sufficient recovery before the next training session. It could therefore be argued that the favourable outcomes seen in Chiang et al. (2007) may not represent the responses that would be seen in an overtrained athlete. Additionally, differences between these studies’ findings could also be due to the use of different DC stimulants i.e., Chiang et al. (2007) used LPS, whereas Mackenzie et al. (2016) used OVA stimulation. It has been shown that the OVA-stimulated and LPS-stimulated DC cytokine responses are different in rodents, with LPS inducing a larger response (Huang et al., 2013). As such, these stimulants may also differently affect the expression of DC cell co-stimulatory molecules and MHC II on DCs upon stimulation.

Mackenzie et al. (2016) found a significant increase in DC CD86 expression, a significant decrease in MHC II expression and no significant differences in CD80 expression in mice who underwent 4 weeks of treadmill running at 6% max velocity for 1 h.d^−1^, 5 days.wk^−1^. DCs transmigrate between peripheral blood and the lymphatic system acting as immune sentinels (Brown et al., 2018). When infection occurs, DCs undergo maturation which involves the upregulation of co-stimulatory molecules CD80 and CD86, the MHC complex and IL-12 cytokine secretion (Wehr et al., 2019). All three of these signals are required for T cell activation, therefore, it is unlikely that an upregulation of one of these signals alone will significantly alter DC function, and ability to induce a T cell response. The discrepancies between findings of these studies (Chiang et al., 2007; Mackenzie et al., 2016), in addition to the lack of studies investigating these DC markers is a cause for expansion upon this preliminary work in rodents towards human models, which becomes increasingly important as exercise training may hold the potential to increase DC maturation, and thus antitumor immunity (Chiang et al., 2007). It is acknowledged however, that much is left to speculation, due to the limited numbers of studies assessing the DC response to intensified training, and those studies that have assessed the DC response, only examined rodent models. Therefore, we cannot make any conclusions in humans, but it does suggest some research in human models is required.

The immune system is a complex system. We cannot assume that periods of intensified training, or the cortisol alterations it may induce, affects all immune markers in the same way. Therefore it would not be unreasonable to see variation in the changes of different immune activation markers with intensified training. Furthermore, resilience is the ability of the body to resist, adapt to, recover or grow in response to stressors (Chow et al., 2022). For example, high levels of immune resilience can reduce illness episodes/hospitalisations and accelerate immune recovery, as shown in COVID-19 patients (Justice et al., 2021). Differences in levels of immune resilience within the participants used in these studies could therefore lead to variations in immune activation and recovery to the same stress, and as such, we cannot assume that all immune systems will respond in the same way to the same level of stressor.

## 5 Conclusion

This review identified numerous immune biomarkers that have been investigated before and after a period of intensified training. Although this review focuses on the normal impact of high intensity training due to the difficulties surrounding confirmation of NFOR/OTS diagnosis, heavy training is a factor involved in the establishment of NFR/OTS, therefore, the results presented could provide evidence that these immune biomarkers are potentially indicative of NFOR/OTS. Results suggest that although some biomarkers indicated significant alterations after a period of intensified training (resting CD8^+^ T Cell and total lymphocyte number, unstimulated IL-1β secretion, and DC CD86 expression), definitive immune biomarkers indicative of the negative states of overtraining are limited. Incompatibilities in methodologies and units of measurements between studies, as well as low study numbers contributed to the inability to identify more definitive immune biomarkers within the literature. This review highlights the need for further research into biomarkers specifically relating to dendritic cells, especially in human models. Additionally, although this review aimed to include females, no study returned from the systematic search controlled for menstrual cycle meaning only male data could be included. Therefore, future research should aim to conduct a controlled study of immune biomarkers in female subjects in response to a period of intensified training in order to widen the applicability of findings. The inclusion of humoral immunity, such as the measure of salivary immunoglobulin A was not considered for this review, however, future reviews should include this as a possible immune biomarker to provide a more holistic overview of the immune state after a period of intensified training. Overall, a period of intensified training has been shown to significantly decrease resting total lymphocyte counts, resting CD8^+^ T cell counts and unstimulated IL-1β levels, and significantly increase DC CD86 expression.

## Data Availability

The original contributions presented in the study are included in the article/Supplementary Material, further inquiries can be directed to the corresponding author.
